# Drug Target Validation of the Protein Kinase *AEK1,* Essential for Proliferation, Host Cell Invasion, and Intracellular Replication of the Human Pathogen Trypanosoma cruzi

**DOI:** 10.1128/Spectrum.00738-21

**Published:** 2021-09-29

**Authors:** Miguel A. Chiurillo, Bryan C. Jensen, Roberto Docampo

**Affiliations:** a Center for Tropical and Emerging Global Diseases, University of Georgia, Athens, Georgia, USA; b Department of Biological Sciences, University of Cincinnati, Cincinnati, Ohio, USA; c Seattle Children’s Research Institute, Seattle, Washington, USA; d Department of Cellular Biology, University of Georgia, Athens, Georgia, USA; University of Manitoba

**Keywords:** CRISPR/Cas9-based mutagenesis, AGC kinase, ATP analog-sensitive, *Trypanosoma cruzi*, cytokinesis

## Abstract

Protein phosphorylation is involved in several key biological roles in the complex life cycle of Trypanosoma cruzi, the etiological agent of Chagas disease, and protein kinases are potential drug targets. Here, we report that the AGC essential kinase 1 (*TcAEK1*) exhibits a cytosolic localization and a higher level of expression in the replicative stages of the parasite. A CRISPR/Cas9 editing technique was used to generate ATP analog-sensitive *TcAEK1* gatekeeper residue mutants that were selectively and acutely inhibited by bumped kinase inhibitors (BKIs). Analysis of a single allele deletion cell line (*TcAEK1-*SKO), and gatekeeper mutants upon treatment with inhibitor, showed that epimastigote forms exhibited a severe defect in cytokinesis. Moreover, we also demonstrated that *TcAEK1* is essential for epimastigote proliferation, trypomastigote host cell invasion, and amastigote replication. We suggest that *TcAEK1* is a pleiotropic player involved in cytokinesis regulation in T. cruzi and thus validate *TcAEK1* as a drug target for further exploration. The gene editing strategy we applied to construct the ATP analog-sensitive enzyme could be appropriate for the study of other proteins of the T. cruzi kinome.

**IMPORTANCE** Chagas disease affects 6 to 7 million people in the Americas, and its treatment has been limited to drugs with relatively high toxicity and low efficacy in the chronic phase of the infection. New validated targets are needed to combat this disease. In this work, we report the chemical and genetic validation of the protein kinase AEK1, which is essential for cytokinesis and infectivity, using a novel gene editing strategy.

## INTRODUCTION

Trypanosoma cruzi, the causative agent of Chagas disease, is a parasitic protist that belongs to the order Kinetoplastida and exhibits a complex life cycle. This parasite has four major developmental stages, epimastigotes and metacyclic trypomastigotes in the insect vector and cell-derived trypomastigotes and amastigotes in the mammalian host, with two replicative stages: the epimastigote and amastigote forms.

Chagas disease affects 6 to 7 million people in the Americas, with over 10,000 deaths annually (https://www.who.int/chagas/en/), and although it is endemic in Latin America, migration has contributed to the globalization of the disease, with more than 300,000 infected people currently residing in the United States (1). There is no vaccine available and, for decades, the treatment of Chagas disease has been limited to two drugs (benznidazole and nifurtimox) with relatively high toxicity and low efficacy for the chronic phase of the infection, in which most of the patients are diagnosed (2–4). Moreover, natural drug resistance has been reported (5, 6).

The approaches to generate new treatments for Chagas disease involve new schemes or strategies for the administration of the available drugs to prevent side effects or searching for new therapeutic candidates to replace them (7). In humans, protein kinases have been a significant focus of drug discovery efforts due to their relevance in cancer, diabetes, inflammation, and neurodegeneration (8). Kinases represent almost 30% of the “druggable human genome,” and approximately 20 protein kinases in the human kinome are targets of FDA-approved inhibitors (8, 9).

Protein phosphorylation plays key roles in intracellular signal transduction pathways and in regulation of many biological processes such as the cell cycle, cell growth, and apoptosis. Phosphoproteomic studies indicate that T. cruzi protein phosphorylation is likely to have an important role in the regulation of the cell cycle, differentiation, metabolism, and survival (10–14). Therefore, protein kinases constitute an attractive class of molecular targets for drug discovery for the treatment of T. cruzi infection.

T. cruzi has approximately 190 eukaryotic protein kinases divided in six groups and an additional group classified as “other” (15). The AGC group of protein kinases, so named for its members cAMP-dependent protein kinase (PKA), cGMP-dependent protein kinase (PKG), and protein kinase C (PKC), includes more than 60 evolutionary related serine/threonine protein kinases in humans (16) and has been considered relatively underrepresented in trypanosomatid genomes. Thirteen genes encoding protein kinases belonging to the AGC group can be found in the T. cruzi genome, which represent about 7% of the kinases of T. cruzi (15, 17), although normalizing to the size of genome, trypanosomatids have about half the AGC kinases of humans (15). Some trypanosomatid AGC kinases, like *AEK1* (named AGC essential kinase 1) (18), cannot be directly assigned to the AGC subfamilies conserved in higher organisms. *AEK1* orthologs in Trypanosoma brucei and Leishmania mexicana, the causative agents of human African trypanosomiasis and cutaneous form of leishmaniasis, respectively, have been described as probably essential in large RNA interference (RNAi) and CRISPR/Cas9 screenings (19–21). Using conditional knockouts, Jensen et al. (18) confirmed that *AEK1* is essential in T. brucei bloodstream forms *in vitro* and *in vivo*. However, no studies have been reported on *AEK1* from T. cruzi.

Most Ser/Thr kinases have a large hydrophobic residue, named the gatekeeper residue, that limits the access to the ATP-binding pocket. This highly conserved amino acid (methionine, leucine, phenylalanine, threonine, etc.) can be mutated, naturally or artificially, to a small residue such as glycine or alanine, creating an extended space within the ATP-binding site (22). The resulting kinase is known as ATP analog-sensitive because the small gatekeeper residue allows that ATP analogs containing a bulky substituent (termed bumped kinase inhibitors or BKIs) complement the shape of the mutant ATP pocket and potentially and specifically inhibit the kinase (23–25). In addition, the ATP analog-sensitive approach has been used in T. brucei to perform functional studies with endogenously expressed small gatekeeper mutants of Polo-like kinase and CRK9 and with conditional knockouts (KOs) of *TbAEK1* (18, 26, 27). To our knowledge, this methodology has not yet been applied to study T. cruzi protein kinases.

In this work, we demonstrate the cytosolic localization of TcAEK1 and the expression levels at different stages of the parasite’s life cycle by analyzing endogenously tagged and overexpressing *TcAEK1* strains. We also generated single knockout mutants (*TcAEK1*-SKO) and used a CRISPR/Cas9-based editing strategy to substitute the *TcAEK1* gatekeeper residue and generate mutants sensitive to known ATP-competitive small molecule inhibitors. ATP analog-sensitive kinase technology has been used as a tool to validate drug targets when no specific inhibitors were available (24). We demonstrate that *TcAEK1* is essential for epimastigote proliferation, trypomastigotes host cell invasion, and amastigote replication. We also show that downregulation or inhibition of *TcAEK1* causes a severe impairment of cytokinesis in the replicative epimastigote form. The engineered *TcAEK1* gatekeeper mutants allowed us to confirm results obtained with *TcAEK1*-SKO cells and to validate *TcAEK1* as a drug target.

## RESULTS

### Expression and localization of the AGC kinase *TcAEK1*.

**FIG 1:**
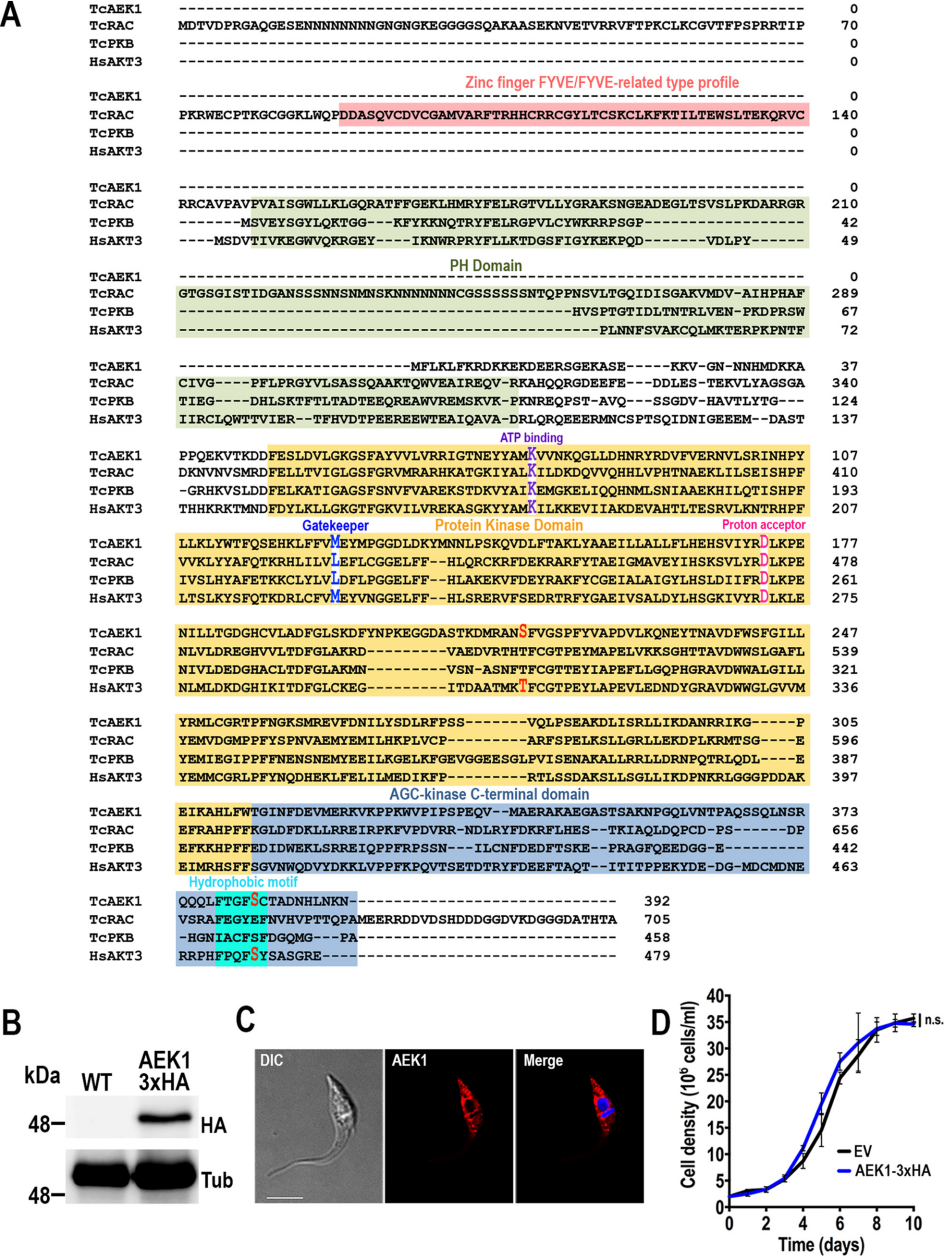
TcAEK1 structure and localization. (A) Amino acid sequence alignment of AGC kinase subfamily members from T. cruzi, TcAEK1, TcRAC, and TcPKB (TriTrypDB IDs: TcYC6_0120630, TcYC6_0106530, TcYC6_0095770, respectively), and human HsAKT3 (GenBank accession number: NP_001357003). Important conserved domains and residues are highlighted. In red are putative conserved phosphorylated amino acid residues. PH, Pleckstrin homology domain. (B) *TcAEK1*-3×HA (AEK1-3×HA) overexpression was confirmed by Western blotting using anti-HA antibodies. Cells transfected with pTREX-n empty vector (EV) were used as the control cell line. Tubulin (Tub) was used as the loading control. (C) IFA showed subcellular localization of TcAEK1-3×HA detected with anti-HA antibodies (AEK1 [red]) in T. cruzi epimastigotes. Merge of red signal and DAPI staining (blue) and DIC (differential interference contrast) images is also shown. Scale bar is 5 μm. (D) Growth of control (EV) and TcAEK1 (AEK1-3×HA) epimastigotes in LIT medium. No significant (n.s.) differences in growth rates were found using one-way ANOVA with multiple comparisons (*n *= 3).

*AEK1* has orthologs in all kinetoplastid species that were found in genome databases. The *AEK1* ortholog of T. cruzi Y strain was found in the TriTryp genome database (http://tritrypdb.org/tritrypdb/, ID: TcYC6_0120630), and it is a single-copy gene composed of 1,179 nucleotides encoding 392 amino acids (∼44.8 kDa). TcAEK1 shares amino acid identity from 68% to 76% (84% to 87% similarity) with the orthologs found in T. brucei and Bodo saltans, respectively. Additionally, full-length TcAEK1 shows 33% identity (58.2% similarity) with human AKT3 (RAC-gamma serine/threonine kinase), reaching 40.7% (65.5% similarity) within the catalytic domain. Interestingly, the annotated ortholog in T. cruzi CL Brener strain genome (Trytrypdb: TcCLB.508479.150) is longer (1,425 nucleotides [nt]) than those found in other T. cruzi strains or kinetoplastid organisms. The open reading frame (ORF) of *TcAEK1* in Y strain starts ∼250 nt downstream of that in CL Brener strain (Fig. S1A). The apparent loss of reading frame in that region occurs in areas of polypyrimidines, and in the absence of confirmation of the molecular weight of the AEK1 protein in CL Brener, the longer N terminus of this strain is likely an annotation error. TcAEK1 shares common features with other members of the AGC protein kinase family, such as the catalytic core and the C-terminal domain, which includes the hydrophobic motif (Fig. 1A). TcAEK1 is not predicted to have a Pleckstrin homology domain (PH) or other N-terminal domain involved in binding biological membranes. Putative phosphorylation sites, which are conserved in AGC kinase proteins to regulate their function, are also predicted in TcAEK1: the first is located in the kinase domain at the “activation loop” (S216, corresponding to HsAKT3 T305), and the second is located in the AGC kinase C-terminal domain (S382, corresponding to HsAKT3 S472) (Fig. 1A).

To determine TcAEK1 cellular localization, we generated a cell line overexpressing the full-length C-terminal triple hemagglutinin (3×HA)-tagged protein (*TcAEK1*-3×HA). Western blotting of epimastigote extracts using anti-HA antibodies showed a major protein band of ∼48 kDa, which is compatible with the predicted mass of the full-length protein plus the 3×HA tag (Fig. 1B). Fluorescence microscopy images showed a cytosolic subcellular localization of the overexpressed-tagged protein. After image deconvolution, TcAEK1 signals exhibited a punctate pattern with an apparent concentration near the nucleus and kinetoplast (Fig. 1C). *TcAEK1-*3×HA had the same growth rate as control cells transfected with the pTREX-n empty vector (Fig. 1D).

### *TcAEK1* expression level is higher in the replicative forms of T. cruzi.

**FIG 2:**
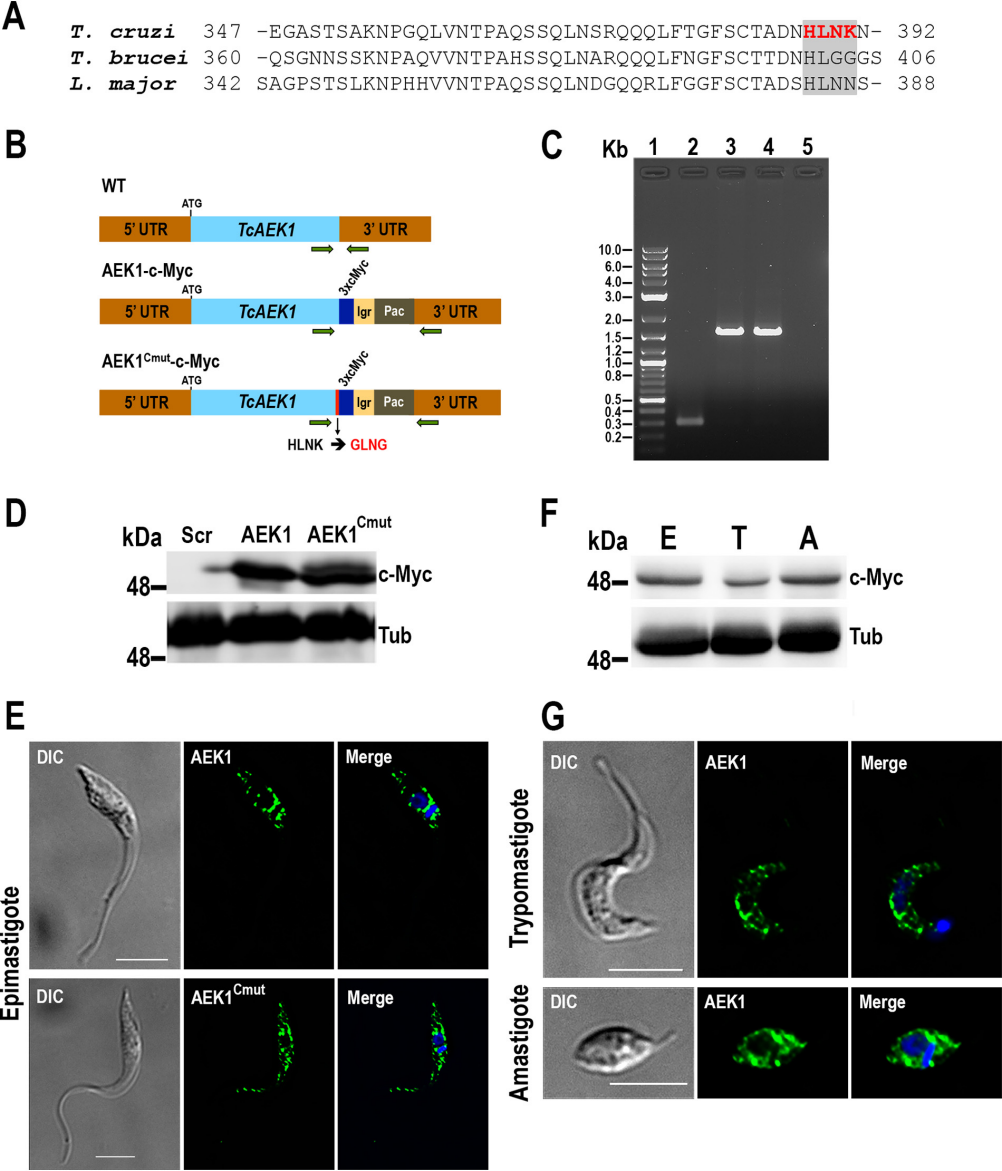
Analysis of expression of endogenously tagged *TcAEK1*-3xc-Myc. (A) Amino acid sequence alignment of the C-terminal region of AEK1 orthologs from T. cruzi, T. brucei, and L. major (TriTrypDB IDs: TcYC6_0120630, Tbg972.3.2430, and LmjF.25.2340, respectively). Enclosed in a gray box, a putative ER retention signal is highlighted in red text (T. cruzi): KKXX-like motif in the C terminus (HLNK) predicted by WoLF PSORT method (https://wolfpsort.hgc.jp/). (B) Schematic representation of *TcAEK1* (WT), endogenously tagged *TcAEK1*-3xc-Myc (AEK1-c-Myc), and *TcAEK1^Cmut^*-3xc-Myc, which has the KKXX-like motif mutated from HLNK to GLNG amino acid residues (AEK1^Cmut^-c-Myc). UTR, untranslated region; Igr, tubulin intergenic region; Pac, puromycin N-acetyltransferase. Horizontal green arrows indicate primers used for tagging verification. (C) PCR verification of integration of DNA donor in the 3′ end of the endogenous *TcAEK1* locus*. TcAEK1* allele size: WT, 300 bp; 3xc-Myc-tagged, 1,581 bp. Lanes: 1, 1-kb plus ladder; 2, scrambled control; 3, *TcAEK1*-3xc-Myc; 4, *TcAEK1^Cmut^*-3xc-Myc; 5, PCR negative control. (D) *TcAEK1* endogenous tagging was verified by Western blotting using anti-c-Myc antibodies (c-Myc). Tubulin (Tub) was used as a loading control. Scr, scrambled; AEK1, *TcAEK1*-3xc-Myc; AEK1^Cmut^, *TcAEK1^Cmut^*-3xc-Myc. (E) Immunofluorescence analysis (IFA) showed localization of endogenously tagged *TcAEK1*-3xc-Myc (upper panel) and *TcAEK1^Cmut^*-3xc-Myc (lower panel) epimastigotes detected with anti-c-Myc antibodies (green). Merge of green signal (AEK1) and DAPI staining (blue) and DIC images are also shown. Scale bars represent 5 μm. (F) Representative Western blotting of TcAEK1-3xc-Myc in total extracts of epimastigotes (column E), tissue culture cell-derived trypomastigotes (column T), and amastigotes (column A) using anti-c-Myc antibodies. Tubulin was used as the loading control. (G) IFA shows localization of endogenously tagged *TcAEK1*-3xc-Myc detected with anti-c-Myc antibodies (green) in trypomastigote and amastigote. Merge of green signal (AEK1) and DAPI staining (blue) and DIC images are also shown. Scale bars represent 5 μm.

The WoLF PSORT tool predicted that the HLNK residues at the C terminus of TcAEK1 could be an endoplasmic reticulum (ER) membrane retention signal: KKXX-like (28) (Fig. 2A). Interestingly, this ER membrane retention signal was not predicted in orthologs from other kinetoplastid organisms. To examine this prediction, we used CRISPR/Cas9 gene editing to generate two endogenously tagged mutant cell lines: one wild-type *TcAEK1* (*TcAEK1*-3xc-Myc) and one with a mutated C terminus (*TcAEK1^Cmut^*-3xc-Myc) (Fig. 2B). *TcAEK1* tagging was confirmed by PCR (Fig. 2C) and also by Western blotting using anti-c-Myc antibodies (Fig. 2D). As shown in the immunofluorescence analysis (IFA) images in Figure 2E, both tagged proteins exhibited subcellular localization pattern similar to that of epimastigotes with the overexpressed *TcAEK1* (Fig. 1C). *TcAEK1^Cmut^*-3xc-Myc epimastigotes did not show a growth defect (not shown). This result suggests that the predicted ER membrane retention signal does not determine the localization of TcAEK1. Furthermore, antibodies against ER (TbBIP) and reservosome (TcCruzipain) markers did not colocalize with TcAEK1 (Fig. S1B and C). Finally, to rule out the possibility that C-terminal tagging (overexpressed or endogenous) affected TcAEK1 localization (i.e., masking the KKXX-like motif), we generated an overexpression cell line with an internal N-terminal Flag tag (*TcAEK1*-Flag) (Fig. S1D and E), which exhibited cellular localization in epimastigote forms similar to that observed with the endogenously C-terminal *TcAEK1-*tagged versions.

We also used the endogenously tagged *TcAEK1*-3xc-Myc cell line to study the expression pattern of *TcAEK1* in the other two developmental stages maintained under laboratory conditions. Initially, TcAEK1-3xc-Myc was detected in cell extracts of epimastigotes, cell-derived trypomastigotes, and amastigotes by Western blotting using anti-c-Myc antibodies (Fig. 2F). As was observed in epimastigotes (Fig. 2E), TcAEK1-3xc-Myc detected with anti-c-Myc antibodies was distributed as punctate pattern and larger aggregates throughout the cytoplasm but concentrated near the nucleus and kinetoplast, in both cell-derived trypomastigotes and amastigotes (Fig. 2G). Therefore, our results confirm the expression of TcAEK1 protein in the three main stages of the T. cruzi life cycle. Densitometry analyses of Western blot experiments, using anti-c-Myc antibodies (Fig. S2), normalized to tubulin protein levels suggest that endogenous TcAEK1 protein levels in cell-derived trypomastigotes were lower than those found in epimastigotes or amastigotes.

### *TcAEK1* knockdown affects growth of epimastigotes and amastigotes and trypomastigote invasion.

**FIG 3:**
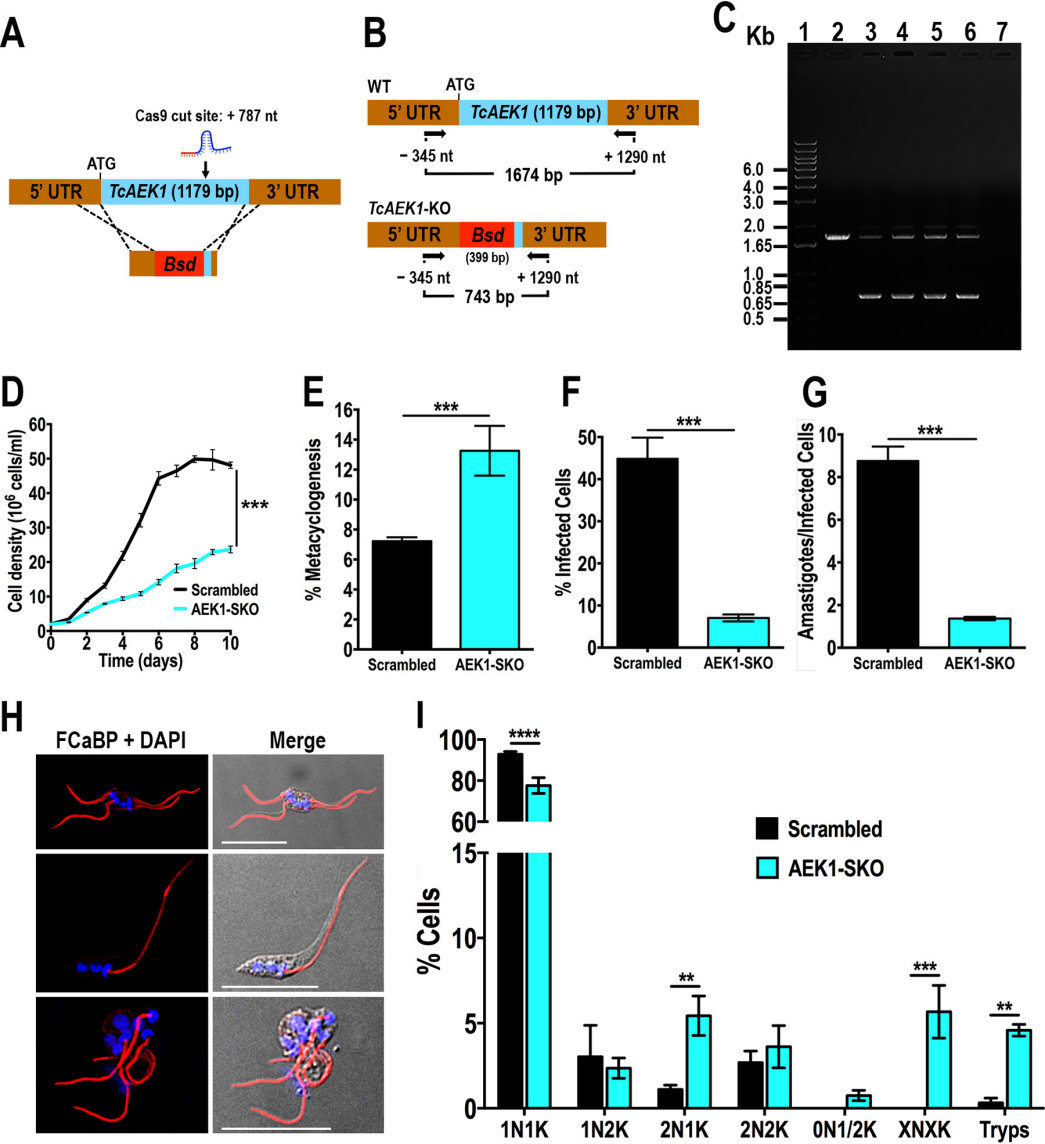
Generation and characterization of *TcAEK1*-SKO cells. (A) Schematic representation of the strategy designed to generate a *TcAEK1*-KO mutant by CRISPR/Cas9-induced homologous recombination. Cas9 introduced a DNA double-strand break at nt +787 of the *TcAEK1* ORF (1,179 bp). DNA was repaired with a blasticidin S-deaminase (*Bsd*) cassette containing 100-bp homologous regions spanning from nt −342 to −243 and from nt +1,094 to +1,190 of the *TcAEK1* locus. (B) PCR primers used to verify *TcAEK1* ablation. Arrows indicate primers added to the reaction. The intact locus generates a PCR product of 1,674 bp, while the disrupted locus generates a DNA fragment of 743 bp. UTR, untranslated region. (C) Only one *TcAEK1* allele was disrupted at its genomic locus in the SKO cell lines. Lanes: 1, 1-kb plus ladder; 2, WT; 3, *TcAEK1-*SKO G418/blasticidin resistant mixed population; 4, *TcAEK1-*SKO clone A3; 5, *TcAEK1-*SKO clone A6; 6, *TcAEK1-*SKO clone B3; 7, PCR negative control. (D) Growth of control (scrambled) and *TcAEK1*-SKO (clone A6) epimastigotes in LIT medium. Student’s *t* test was applied to growth rates calculated from each growth curve (*n *= 3; ***, *P < *0.001). (E) Percentage of metacyclic trypomastigotes in epimastigote cultures after incubation in TAU 3AAG medium. Scrambled and *TcAEK1*-SKO (clone A6) epimastigote differentiation to metacyclic trypomastigotes was quantified by staining with DAPI to distinguish the position of the kinetoplast by fluorescence microscopy. (F and G) Scrambled and *TcAEK1*-SKO (clone A6) trypomastigote infection of Vero cells. There were significant differences in the percentage of infected Vero cells at 4 h postinfection (F) as well as in the number of intracellular amastigotes per infected host cell observed 48 h postinfection (G) by *TcAEK1*-SKO mutant cells. In panels E, F, and G, values are means ± SD (*n *= 3), ***, *P < *0.001 by Student’s *t* test. (H) IFA of representative cytological abnormalities observed in *TcAEK1*-SKO epimastigote culture. Flagella were detected with monoclonal anti-TcFCaBP antibodies (red), and DAPI was used to stain kinetoplast and nuclear DNA (blue). DIC (differential interference contrast) images are also shown. Scale bars represent 15 μm. (I) Effect of single *TcAEK1* gene KO on nucleus/kinetoplast patterns established after DNA labeling with DAPI. At least 200 cells were counted for each analysis. Data represent means, and error bars indicate SD from 3 independent experiments. XNXK, X > 2; Tryps, metacyclic trypomastigotes. **, *P < *0.01; ***, *P < *0.001; ****, *P < *0.0001 (two-way ANOVA with Sidak’s multiple-comparison test).

To explore the role of *TcAEK1*, we designed a CRISPR/Cas9 strategy to generate knockout mutants of this gene (Fig. 3A). The method, which has been successfully adapted recently to T. cruzi (29–33), involves the constitutive expression of Cas9 and specific single guide RNA (sgRNA) and the utilization of a template cassette to promote double-strand break repair by homologous-directed repair (HDR). After several attempts cotransfecting epimastigotes with the sgRNA/Cas9-harboring plasmid (using two different sgRNA separately) together with a DNA donor, we were unable to obtain drug-resistant stable transfectants. Therefore, we followed a two-step strategy: first, we obtained a cell line that constitutively expresses Cas9 and *TcAEK1-*sgRNA-787, and later, these epimastigotes were further transfected with the blasticidin resistance cassette. Subsequently, we obtained clonal populations from stable transfectants by limiting dilution. We confirmed by PCR that only one *TcAEK1* allele was replaced by the DNA donor cassette at the specific loci (Fig. 3B and C) in all clonal populations obtained. Another attempt following the same approach but using a different DNA donor cassette also resulted in a single *TcAEK1* allele eliminated (Fig. S3A to C).

We then followed a third strategy to generate *TcAEK1* null mutants, which was designed to disrupt the wild-type (wt) allele with a puromycin resistance cassette in a *TcAEK1*-SKO clonal population obtained in the second attempt (Fig. S4A). First, we confirmed that the protospacer and protospacer-adjacent motif (PAM) sequence for *TcAEK1-*sgRNA-787 was intact at the wt allele and that Cas9-GFP was expressed (Fig. S4B and C). After transfection and selection, genomic DNA (gDNA) of G418/blasticidin/puromycin-resistant stable populations were analyzed by PCR, revealing the presence of three bands whose sizes corresponded to those predicted for the wt *TcAEK1* allele and those disrupted by the blasticidin and puromycin cassettes (Fig. S3D and E). This result suggests that *TcAEK1*-SKO cells probably amplified the *TcAEK1* locus because null alleles can have lethal effects in epimastigotes.

We therefore studied the effects of the *TcAEK1* hemizygous deletion or single knockout (SKO) on proliferation and infectivity of T. cruzi. *TcAEK1*-SKO epimastigotes exhibited a growth rate in liver infusion tryptose (LIT) medium significantly lower than that of parasites transfected with a scrambled sgRNA (Fig. 3D), providing evidence that *AEK1* is important for epimastigote proliferation. Moreover, the ability of *TcAEK1*-SKO epimastigotes to differentiate *in vitro* into metacyclic trypomastigotes (metacyclogenesis in triatome artificial urine [TAU] 3AAG medium) was significantly higher compared to that of control cells (Fig. 3E). We also investigated the ability of trypomastigotes to infect tissue-cultured cells. Both invasion of host cells and replication of intracellular amastigotes were significant impaired in *TcAEK1*-SKO (Fig. 3F and G). These results together with the failure in obtaining *TcAEK1* null mutants suggest that this gene is essential in T. cruzi.

*TcAEK1*-SKO epimastigotes with a severe growth defect were observed under fluorescence microscopy using anti-TcFCaBP antibodies to stain the flagella. Approximately 10 to 15% of *TcAEK1*-SKO cells were larger and showed morphological alterations, as well as an aberrant number of nuclei/kinetoplasts and flagella (Fig. 3H, see also Fig. S3D). To further examine the growth defect phenotype upon knockdown of *TcAEK1*, we quantified the number of nuclei and kinetoplasts per cell after DAPI (4′,6-diamidino-2-phenylindole) staining in control and *TcAEK1*-SKO epimastigotes cultured in LIT medium (Fig. 3I). Nuclear/kinetoplast content (N/K) in T. cruzi epimastigotes progresses during its normal cell cycle from 1N1K to 1N2K to 2N2K (34). The kinetoplast divides before the nucleus, and mitosis of 1N2K yields 2N2K cells that divide during cytokinesis into two 1N1K cells. In normal nonsynchronous, logarithmic-phase epimastigote cultures, most of the cells have 1N1K content (∼80 to 95%) (34). Compared with those of control cells, *TcAEK1*-SKO epimastigotes stained with DAPI exhibited an increase of binucleated cells, with cell numbers of 2N1K significantly higher. We also observed a significant accumulation of multinucleated cells (XNXK, X > 2), suggesting a defective cytokinesis. Interestingly, *TcAEK1*-SKO epimastigote cultures in LIT medium exhibited nearly 5% of cells (*P < *0.01) with morphology compatible with that of metacyclic trypomastigotes.

### Design and analysis of ATP analog-sensitive TcAEK1.

**FIG 4:**
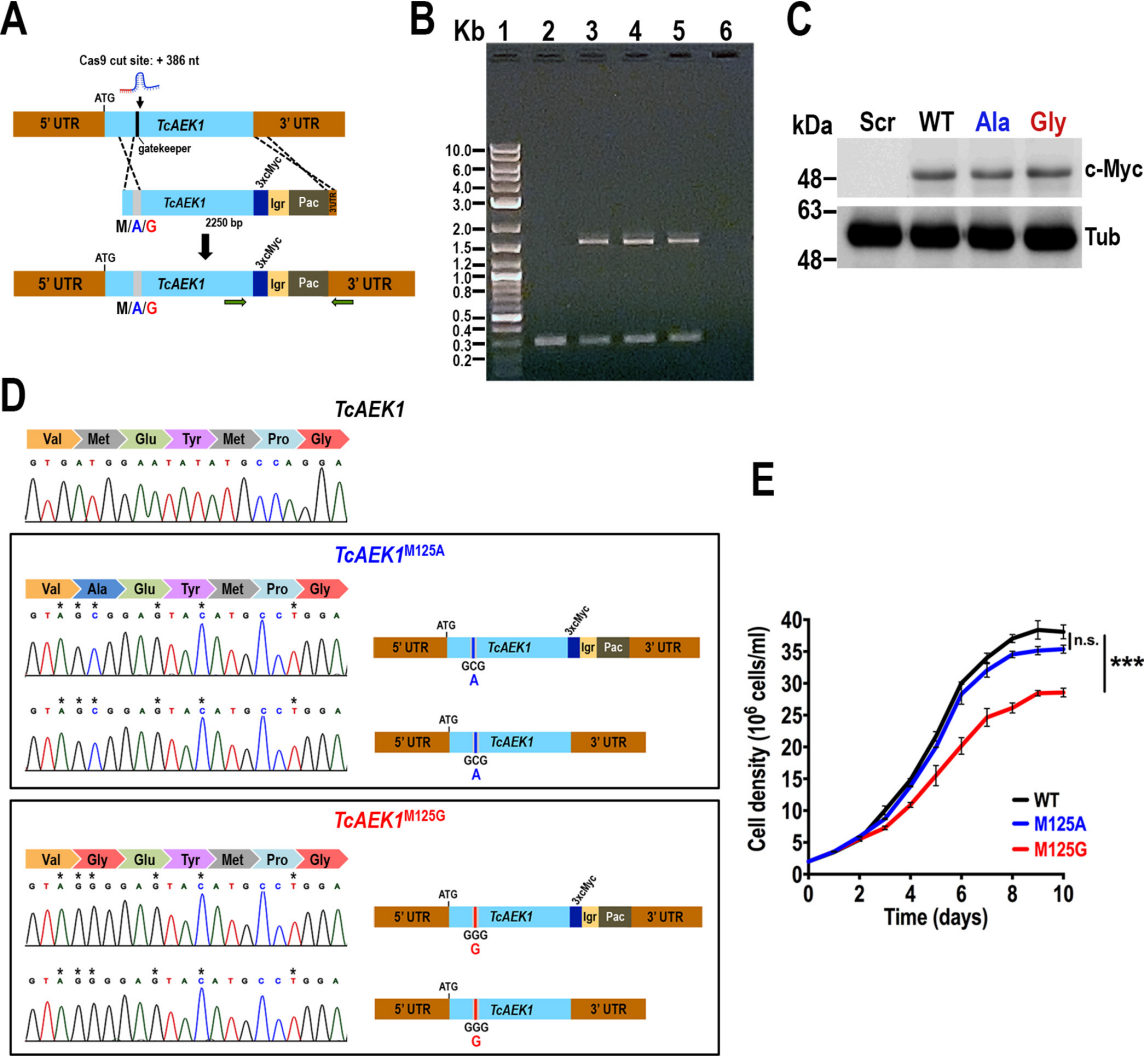
Generation of analog-sensitive *TcAEK1.* (A) Schematic representation of CRISPR/Cas9-mediated strategy for *TcAEK1* gatekeeper residue knock-in and C-terminal endogenous tagging. Cas9 introduced a DNA double-stranded break at nt +386 of the *TcAEK1* ORF. Homologous-directed repair was induced cotransfecting epimastigotes with the DNA donor cassette, containing homologous regions to *TcAEK1* (light blue) and to the *TcAEK1* 3′ UTR (light brown). The HDR determines integration of 3xc-Myc and antibiotic resistance gene (*Pac*, puromycin N-acetyltransferase) at the 3′ end of *TcAEK1*, as well as the mutation of the *TcAEK1* gatekeeper residue codon sequence, which is included in the protospacer, as well as other silent mutations in the protospacer sequence avoiding being targets of the constitutively expressed Cas9. UTR, untranslated region; Igr, tubulin intergenic region. We used 3 different DNA donor cassettes to maintain the methionine in the wild-type *TcAEK1* gatekeeper residue (M125) or to mutate it to the smaller alanine (M125A) or glycine (M125G) residues. M, M125; A, M125A; G, M125G. Horizontal green arrows indicate primers used for tagging verification. (B) PCR verification of cassette integration into *TcAEK1* locus. *TcAEK1* allele size: WT, 300 bp; 3xc-Myc-tagged, 1,581 bp. Lanes: 1, 1-kb plus ladder; 2, scrambled control; 3, *TcAEK1*^WT^-3xc-Myc; 4, *TcAEK1*^M125A^-3xc-Myc; 5, *TcAEK1*^M125G^-3xc-Myc; 6, PCR negative control. (C) *TcAEK1* endogenous tagging in gatekeeper residue mutants was verified by Western blotting using anti-c-Myc antibodies (c-My). Tubulin (Tub) was used as a loading control. Scr, scrambled, WT, *TcAEK1*^WT^-3xc-Myc; Ala, *TcAEK1*^M125A^-3xc-Myc; Gly, *TcAEK1*^M125G^-3xc-Myc. (D) *TcAEK1* gatekeeper *in situ*-mutated nucleotide sequence was confirmed in PCR-amplified wild-type and tagged size DNA fragments. Electropherograms show that, despite that only one allele was tagged at its C terminus, both alleles were mutated in each *TcAEK1* gatekeeper mutant cell line: *TcAEK1*^M125A^ and *TcAEK1*^M125G^. Asterisks (*) indicate mutations generated in the protospacer sequence. (E) Growth of control *TcAEK1*^WT^ (WT), *TcAEK1*^M125A^ (M125A), and *TcAEK1*^M125G^ (M125G) epimastigotes in LIT medium. One-way ANOVA with multiple comparisons was applied to growth rates calculated from each growth curve (*n *= 3, ***, *P < *0.001).

To gain more insight into the TcAEK1 protein function *in vivo* and to chemically validate it as a potential drug target, we generated *TcAEK1* mutants specifically susceptible to ATP analogs that behave as BKIs. We designed a CRISPR/Cas9 mutagenesis strategy for mutation of the gatekeeper residue (methionine 125; M125) to a small residue (alanine or glycine) conferring specific sensitivity to BKIs. The method used consisted of cotransfecting T. cruzi wt epimastigotes with the sgRNA/Cas9-harboring plasmid plus a linear DNA donor (2,250 bp), which promotes HDR and introduces the gatekeeper mutation, a 3xc-Myc-tag to verify the expression of mutated *TcAEK1* by Western blotting, and the *Pac* gene to allow selection of antibiotic-resistant mutants (Fig. 4A). After G418/puromycin selection, we obtained clonal populations for both *TcAEK1* gatekeeper residue mutants (*TcAEK1*^M125A^ and *TcAEK1*^M125G^). To confirm the *TcAEK1* tagging, we extracted gDNA and performed PCRs with specific primers, and in all clones obtained we detected a single tagged allele (Fig. 4B). Expression of the *TcAEK1* tagged allele in the three mutant cell lines (*TcAEK1*^WT^, *TcAEK1*^M125A^, and *TcAEK1*^M125G^) was verified by Western blotting using anti-c-Myc antibodies (Fig. 4C), and Sanger sequencing confirmed that both alleles had gatekeeper residue mutated *TcAEK1*^M125A^ (two clones) and *TcAEK1*^M125G^ (one clone), in addition to including the silent mutations introduced in the protospacer sequence (Fig. 4D). Thus, these results indicate that in *TcAEK1*^M125A^-3xc-Myc and *TcAEK1*^M125G^-3xc-Myc cell lines, the HDR mechanism repaired the *TcAEK1* normal allele just in the region close to the Cas9/sgRNA cleavage site, probably using the mutated *TcAEK1* allele (tagged) as the DNA donor. *TcAEK1*^WT^ and *TcAEK1*^M125A^ epimastigotes exhibited similar growth rates in a rich medium (LIT), whereas *TcAEK1*^M125G^ revealed a growth rate significantly lower than that of the control (Fig. 4E).

### Effects of BKIs on morphology and viability of ATP analog-sensitive *TcAEK1* gatekeeper mutant epimastigotes and metacyclic trypomastigotes.

To explore the sensitivity of the ATP analog-sensitive *TcAEK1* gatekeeper mutants to BKIs, we performed a pilot experiment growing cells in increasing concentrations of the BKI compounds 1294 and 1553 (Fig. S5A and B). BKI-1294 and its derivative BKI-1553 have been used to specifically inhibit *in vitro* and *in vivo* calcium-dependent protein kinase (CDPK) family members in apicomplexan parasites and the ATP analog-sensitive-AEK1 of T. brucei (18, 35–39). Cells were observed under light microscopy after they were incubated for 48 h in LIT medium. Rounded abnormal forms were present in BKI-1294- or BKI-1553-treated cultures of both mutant cell lines *TcAEK1*^M125A^ (M125A) and *TcAEK1*^M125G^ (M125G). At high concentrations of BKIs (>5 μM), cells were fragmented in a dose-dependent manner, while cells were morphologically intact in control *TcAEK1*^WT^ (WT) incubated with BKIs and in M125A and M125G mutants incubated with 0.2% dimethyl sulfoxide (DMSO).

**FIG 5:**
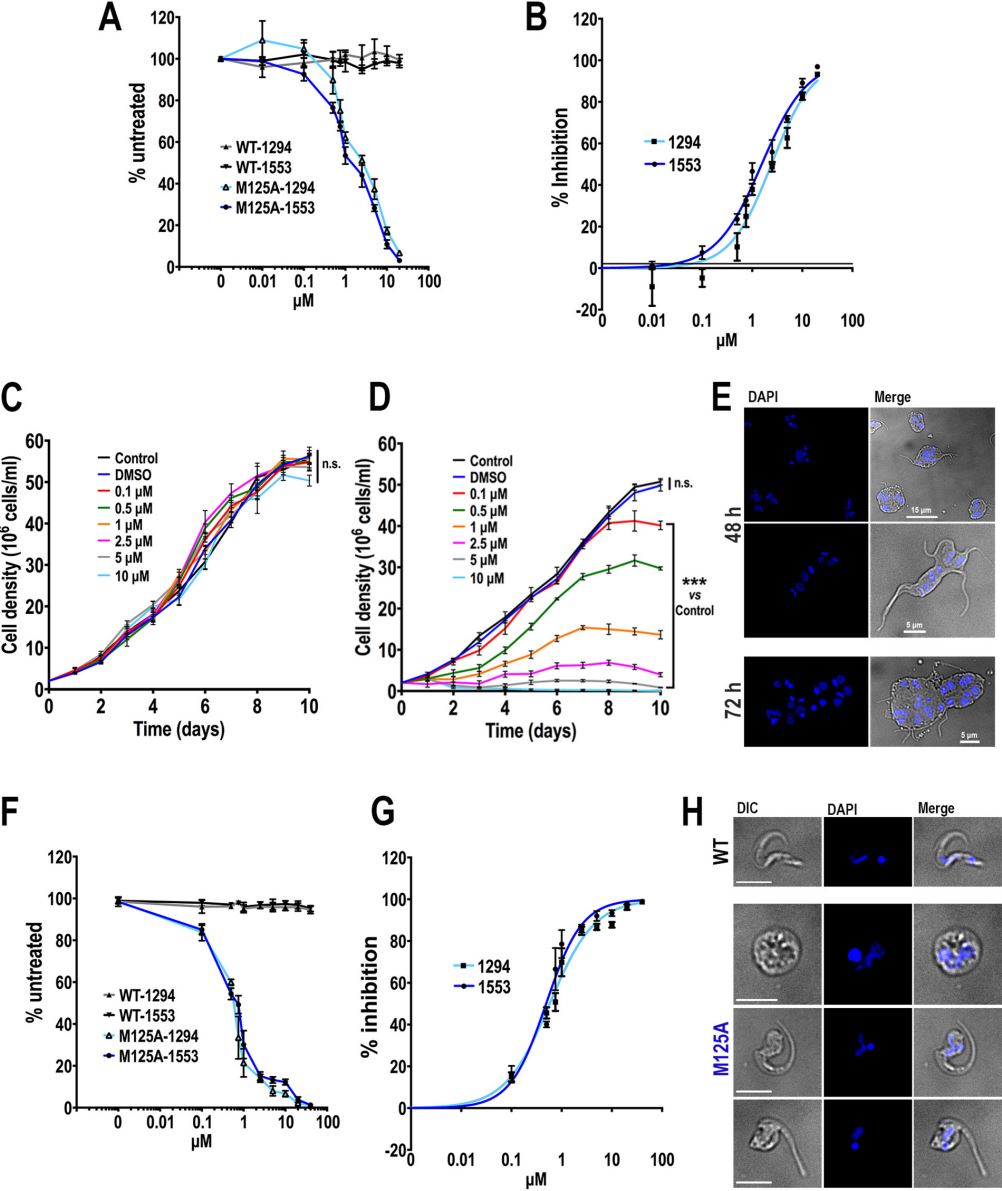
Effect of BKI-1553 on *TcAEK1* gatekeeper mutant epimastigotes and metacyclic trypomastigotes. (A) Cell viability of *TcAEK1*^WT^ (WT) and *TcAEK1*^M125A^ (M125A) epimastigotes incubated for 48 h with various drug (1294 or 1553) concentrations in LIT medium. Cell viability was monitored with the alamarBlue assay. Values were compared with the same cell line in the absence of the inhibitor (set as 100%). (B) Dose response of BKIs 1294 or 1553 against *TcAEK1*^M125A^ epimastigotes. Values in panels A and B are means ± SD of 2 independent experiments, each done in triplicate. (C and D) Growth of *TcAEK1*^WT^ (C) and *TcAEK1*^M125A^ (D) epimastigotes in LIT medium incubated with different concentrations of compound 1553. One-way ANOVA with multiple comparisons was applied to growth rates calculated from each growth curve compared with that of the control (*n *= 3, n.s., not significant, ***, *P < *0.001). (E) Representative images displaying aberrant cell morphology and DNA content of *TcAEK1*^M125A^ epimastigotes incubated with 1.5 μM compound 1553 for 48 h and 72 h. DAPI staining (blue) and merge with DIC images are shown. Scale bars are shown. (F) Cell viability of *TcAEK1*^WT^ (WT) and *TcAEK1*^M125A^ (M125A) metacyclic trypomastigotes incubated for 24 h with various BKI (1294 or 1553) concentrations in LIT medium. Values were compared with the same cell line in the absence of the inhibitor (set as 100%). (G) Dose response of BKIs 1294 or 1553 against *TcAEK1*^M125A^ metacyclic trypomastigotes. For panels F and G, number of parasites was counted in a Neubauer chamber and death percentage was estimated relative to control treatment (0.1% DMSO). Values plotted are means ± SD of 2 independent experiments, each done in triplicate. (H) Representative images of *TcAEK1*^WT^ (WT) and *TcAEK1*^M125A^ (M125A) metacyclic trypomastigotes incubated with 0.5 μM compound 1553 for 24 h. DAPI staining (blue) and merge with DIC images are shown. Scale bars represent 5 μm.

Based on the fact that the effect of the BKIs on both mutants is apparently similar but the *TcAEK1*^M125G^ mutant grows more slowly, we focused on parasites expressing *TcAEK1*^WT^ or the *TcAEK1*^M125A^ mutant. The reduction of alamarBlue by metabolically active cells was used as a viability indicator. As seen in Figure 5A, parasites expressing wt *TcAEK1* (WT) were not affected by up to 20 μM either BKI-1294 or BKI-1553. In contrast, survival of parasites expressing *TcAEK1*^M125A^ mutant (M125A) was greatly decreased in the presence of BKI-1294 or BKI-1553 in a dose-dependent manner starting at 0.5 μM. The 50% inhibitory concentration (IC_50_)/48 h value of compounds BKI-1294 and BKI-1553 for *TcAEK1*^M125A^ epimastigotes was 2.42 ± 0.35 μM and 1.55 ± 0.15 μM, respectively (Fig. 5B). BKI-1553 was chosen for further study because of its lower IC_50_ for *TcAEK1*^M125A^ epimastigotes. A representative set of growth curves of wt and *TcAEK1*^M125A^ epimastigotes, in the presence of various concentrations of BKI-1533 (100 nM to 10 μM), is shown in Figure 5C and D, respectively. No differences were found in the growth rates of *TcAEK1*^WT^ cells in all ranges of concentrations compared with those of epimastigotes cultured without drug (control) (Fig. 5C). On the contrary, *TcAEK1*^M125A^ cells exhibited a significant dose-dependent decrease of growth rates in the presence of BKI-1553, whereas parasites in the presence of solvent (0.1% DMSO) grew normally (Fig. 5D), confirming the importance of *TcAEK1* for epimastigote proliferation. After 48 h in culture with 1.5 μM BKI-1553, DAPI-stained *TcAEK1*^M125A^ cells were large, pleomorphic in shape, and multinucleated and had multiple attached flagella (Fig. 5E).

We were unable to test BKI compounds in host cell invasion assays because the *TcAEK1* gatekeeper mutants have severely reduced ability to infect Vero cells, even though *TcAEK1*^M125A^ cells exhibited no defects in *in vitro* metacyclogenesis compared with *TcAEK1*^WT^ cells (Fig. S6).

We next incubated metacyclic trypomastigotes purified by DEAE cellulose columns with BKI-1294 or BKI-1553 (100 nM to 40 μM). Those expressing *TcAEK1*^M125A^ showed a drastic reduction of viability of metacyclic trypomastigotes upon BKI treatment in a dose-dependent manner (Fig. 5F), while *TcAEK1*^WT^ metacyclic cells remained fully viable in the presence of the BKIs. The estimated IC_50_ value (IC_50_/24 h) of BKI-1294 or BKI-1553 for *TcAEK1*^M125A^ metacyclic trypomastigotes was 578 ± 72 nM and 421 ± 76 nM, respectively (Fig. 5G). As shown in Figure 5H, *TcAEK1*^M125A^ metacyclic trypomastigotes incubated for 24 h with BKI-1553 exhibited severe morphological alterations. This last set of results indicates that *TcAEK1* also plays an important role in this nonreplicative stage of T. cruzi.

### Gatekeeper mutants treated with BKI confirmed that *TcAKE1* is required for cytokinesis in the epimastigote forms of T. cruzi.

**FIG 6:**
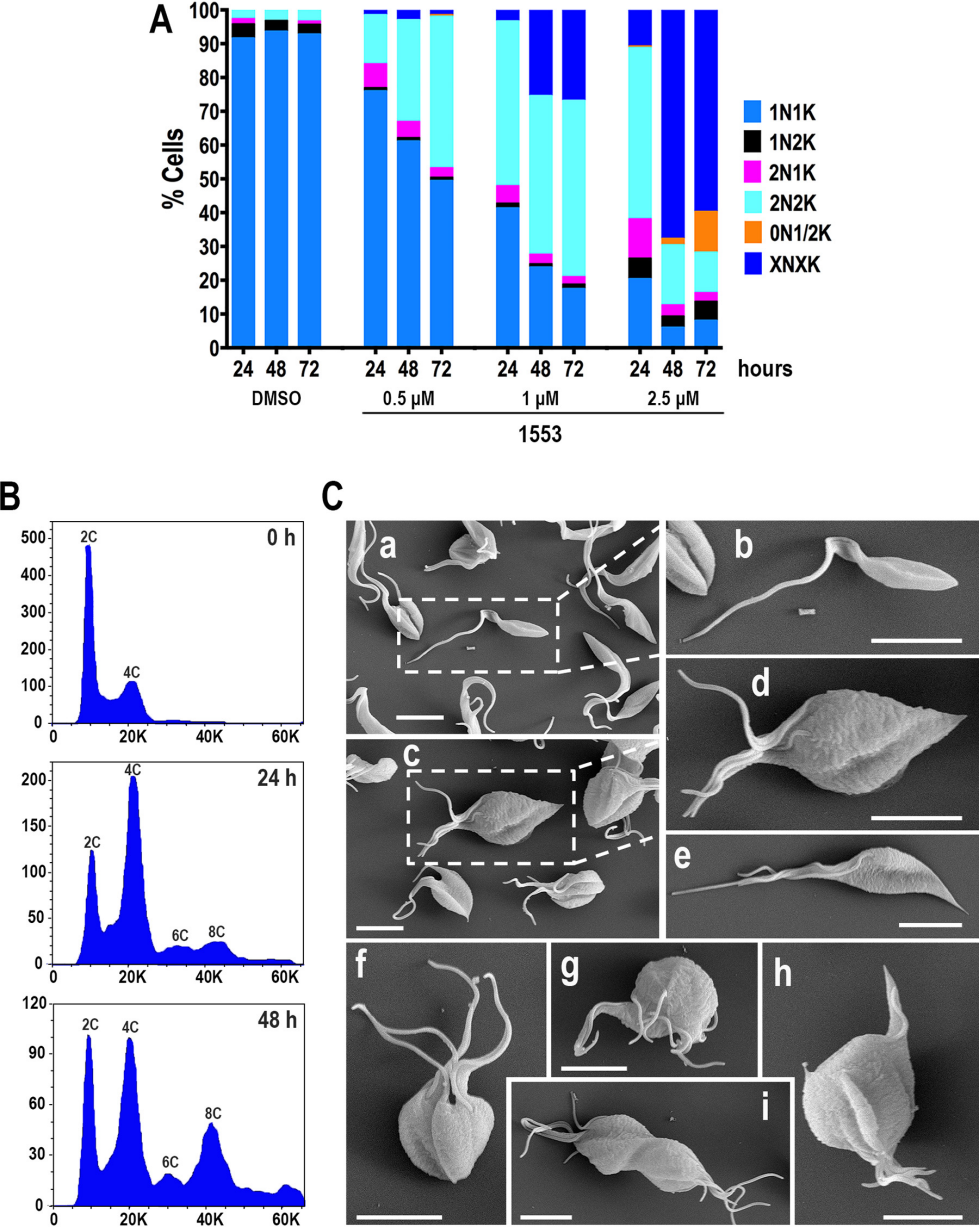
Incubation of ATP analog-sensitive *TcAEK1*^M125A^ epimastigotes with BKI 1553 leads to cell cycle and morphological alterations. (A) Kinetoplast/nucleus configuration of *TcAEK1*^M125A^ epimastigotes, which were treated with 0, 0.5, 1.0, or 2.5 μM compound 1553, was quantified by DAPI staining at 24, 48, and 72 h. Representative of three experiments (*n *= 200 cells). XNXK, X > 2. (B) Flow cytometry analysis of *TcAEK1*^M125A^ epimastigotes incubated with 1.5 μM compound 1553. Analysis was made at 0 h (upper panel), 24 h (middle panel), and 48 h (bottom panel) of incubation with drug. The positions of cells having 2C DNA content (diploid), 4C, 6C, and 8C are indicated. (C) Representative images of scanning electron microscopy of control *TcAEK1*^WT^ (a, b) and *TcAEK1*^M125A^ parasites cultured in the presence of 1.5 μM compound 1553 for 48 h (c to i). Note that analog-sensitive *TcAEK1*^M125A^ shows a multiflagellated and rosette-like morphology.

We also evaluated the N/K pattern of *TcAEK1*^M125A^ epimastigotes cultured in the presence of BKI-1553. Figure 6A shows a decrease in 1N1K cells and an increase in binucleated (2N2K and 2N1K) and multinucleated cells (XNXK, X > 2) depending on the drug concentration and incubation time. These data are consistent with defective cytokinesis and suggest a failure in cell cycle progression in parasites with inhibited *TcAEK1*. Additionally, cells containing 1 or 2 kinetoplasts (termed a zoid) were observed at 48 and 72 h of incubation with the higher concentration of BKI-1553 tested (2.5 μM), suggesting that under these conditions the inhibition of *TcAEK1*^M125A^ can impair the cell cycle in other points or cause nuclear mis-segregation or degradation. Cell DNA content in *TcAEK1*^M125A^ epimastigotes was determined by flow cytometry at 0, 24, and 48 h of incubation with BKI-1553 (Fig. 6B). At the beginning of the experiment (0 h of incubation), *TcAEK1*^M125A^ epimastigotes showed a typical cell cycle curve, having a predominant G_0_/G_1_ population (DNA content, 2C) with relatively fewer cells in S phase and G_2_/M (DNA synthesis and 4N DNA content, respectively) (Fig. 6B, upper panel). The number of tetraploid (4C, G_2_/M) and polyploid (6C and 8C) cells was abnormally elevated at 24 h of treatment (Fig. 6B, middle panel). After 48 h of incubation, *TcAEK1*^M125A^ cells exhibited a predominance of polyploidy with an increase in the proportion of higher-DNA-content cells (Fig. 6B, bottom panel), which is consistent with a defect in cytokinesis.

### Electron microscopy analysis of ATP analog-sensitive *TcAEK1* treated with BKIs confirms cell division defects.

**FIG 7:**
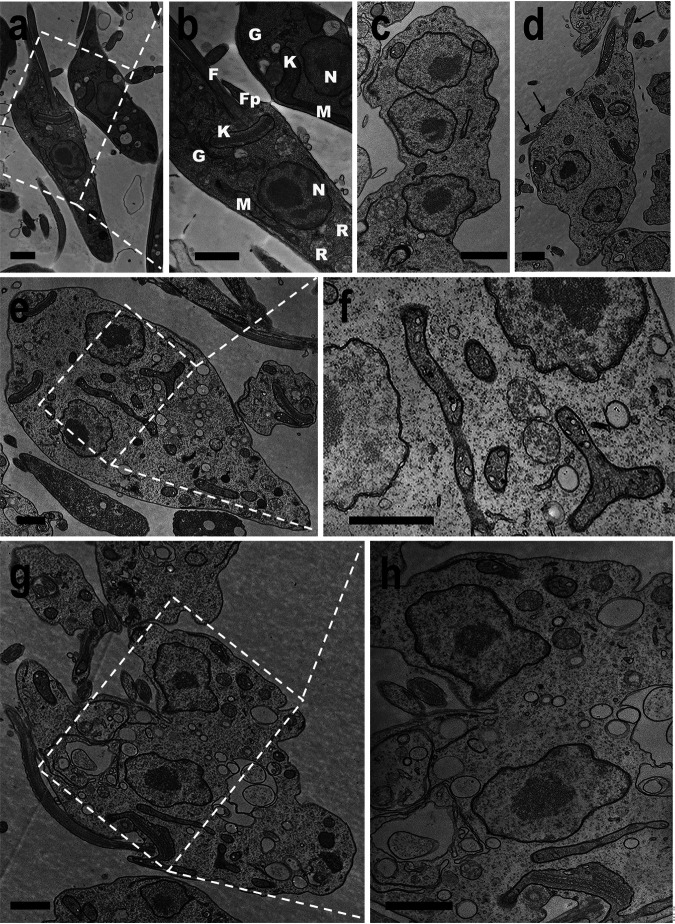
Transmission electron microscopy of T. cruzi cells treated with BKI 1553. Control *TcAEK1*^WT^ (a, b) and *TcAEK1*^M125A^ parasites were treated with 1.5 μM compound 1553 for 48 h (c to h). Arrows indicate multiple flagella of rosette-like cells (d). Cytosolic vacuolization can be observed in g and h. N, nucleus; K, kinetoplast; F, flagellum; Fp, flagellar pocket; R, reservosome; G, Golgi complex; M, mitochondrion. Bars represent 1 μm.

Figure 6C shows scanning electron microscopy (SEM) pictures of *TcAEK1*^WT^ (panels a and b) and *TcAEK1*^M125A^ cells (panels c to i) after treatment with BKI-1553 for 48 h. Most *TcAEK1*^M125A^ cells are arrested in cytokinesis exhibiting a rosette-like or “pumpkin-like” morphology bearing several cell bodies connected. These aggregates of cells show multiple flagella attached, and in some cases (panels c, d, and f), each component still retains the shape of normal cells bound at the posterior end. Parasites did not have, or showed only an incipient, anterior cleavage furrow and longitudinal invagination, but these did not progress. These images are compatible with those observed under fluorescence microscopy (Fig. 5E) and also with DNA content analyzed by flow cytometry (Fig. 6B). Transmission electron microscopy (TEM) cross sections of *TcAEK1*^M125A^ epimastigotes treated for 48 h confirmed that cell aggregates corresponded to a single massive undivided cell (Fig. 7c to h). In general, it is possible to distinguish the cell components of the multinuclear bodies and associated peripheral flagella. Large vacuoles with retracted content and double membrane in cytokinesis-arrested cells could correspond to autophagosomes (Fig. 7g and h). Some mitochondria of aggregate cells have dilated cristae (Fig. 7e and f). Therefore, we stained live *TcAEK1*^M125A^ cells treated with BKI-1553 with MitoTracker. A net-like mitochondrial staining compatible with undivided cell bodies was observed (Fig. S7). Interestingly, MitoTracker fluorescence of treated *TcAEK1*^M125A^ also suggests no obvious defect in mitochondrial membrane potential.

## DISCUSSION

Our studies genetically and chemically validated TcAEK1 as an essential protein in T. cruzi. We found that parasites hemizygous for *AEK1* and those in which AEK1 was specifically inhibited through a chemical genetic approach showed similar phenotypes. A single KO of *TcAEK1* resulted in slowed growth of the replicative stages of the parasite and inhibition of cell-derived trypomastigote invasion of host cells. Both *TcAEK1*-SKO and mutants susceptible to inhibition by BKIs showed similar effects on cytokinesis, as revealed by the appearance of multinucleated cells with several flagella. Metacyclic forms susceptible to BKIs also underwent a severe loss of viability when exposed to those inhibitors, suggesting that TcAEK1 is also important for the nonreplicative stages of the parasite.

In this work, we generated T. cruzi cell lines that exclusively express ATP analog-sensitive TcAEK1 mutants by adapting a CRISPR/Cas9-based mutagenesis strategy that we used previously (40). It is known that the space-creating mutation at the ATP-bonding pocket of kinases is often functionally silent. However, some kinases can suffer variable degrees of activity impairment (25, 41). It is known that there are no mammalian kinases with small gatekeeper residues (25), while that is not the case in apicomplexan parasites, and this has been exploited to perform functional studies and to test selective inhibitors (35, 37, 42–49). Interestingly, we found in the T. cruzi Y strain genome one protein kinase that possesses a small gatekeeper residue (TriTrypDB ID: TcYC6_0069280, residue A1141), which is annotated as STE/STE11 Ser/Thr-protein kinase, although no functional data are available on this putative kinase. The correlation in the phenotype between *TcAEK1*^M125A^ in the presence of BKIs and the *TcAEK1*-SKO, combined with the lack of any phenotype in edited strains expressing tagged wild-type alleles upon exposure to BKI, indicates that there was a selective and specific effect of the inhibitors against the engineered TcAEK1.

Our data indicate that TcAEK1 appears to be required for epimastigote entry into cytokinesis after cells replicated their DNA and underwent mitosis. Cytokinesis is a critical step in the cell division cycle with multiple pathways and various degrees of redundancy that are monitored by specific signaling of checkpoints. This process in trypanosomatids possesses some complexities and peculiar features related to their complex life cycle, developmental stages which are morphologically different and highly polarized, and the need to replicate and segregate a series of single-copy organelles. Moreover, in these organisms, cytokinesis has been considered a microtubule-focused mechanism (50, 51). It has been described in T. brucei that two evolutionarily conserved protein kinases, the Polo-like kinase and the Aurora B kinase, and a group of trypanosome-specific proteins, including the cytokinesis initiation factors CIF1 to CIF4 and CIF1-interacting proteins FRW1 and FPRC, predominantly regulate control of cytokinesis initiation (52–57). Furthermore, Jones et al. (19) identified several protein kinases as essential for cytokinesis in bloodstream forms in their high-throughput RNAi screening.

Knowledge about cytokinesis and its regulation in T. cruzi lags far behind that in T. brucei, since only a few genes have been associated with this biological process in the first parasite. Thus, in T. cruzi, three Aurora kinase homologues were identified (*TcAUK1* to *3*), being *TcAUK1* functional homologues to human Aurora B kinase and related to mitotic spindle assembling, chromosome segregation, and the initiation of kinetoplast duplication (58). Deletion of *TcKMP*-11 (kinetoplastid membrane protein-11) resulted in a substantial population of epimastigotes with cytokinesis defects (59). Furthermore, T. cruzi epimastigotes overexpressing high mobility group B protein (TcHMGB) exhibited impaired cytokinesis, suggesting a role in DNA replication and cell cycle progression control (60). Finally, inhibition of casein kinase 2, for which interaction with tubulin has been established, also triggered blockage of cytokinesis (61).

The essentiality and importance in cytokinesis of *AEK1* were previously assessed in T. brucei. In a high-throughput RNAi-based phenotyping approach, bloodstream forms exhibited a significant loss of fitness after 6 days of RNAi targeting *TbAEK1* (21). Moreover, Jones et al. (19), using a T. brucei kinome-wide RNAi library that included 190 predicted protein kinases, observed that knockdown of *TbAEK1* induced a severe defect in cytokinesis and cell death in bloodstream forms. After induction of RNAi, cultures exhibited accumulation of cells with multiple nuclei and kinetoplasts (>2N > 2K cells). Essentiality of *TbAEK1* in bloodstream forms was subsequently confirmed using conditional KOs and RNAi experiments (18), demonstrating that *TbAEK1* knockdown caused reduced growth, defective cytokinesis, and cell death. Jensen et al. (18) also generated ATP analog-sensitive mutants in which the gatekeeper site was mutated to alanine or glycine, and they observed that BKI-1294 treatment of mice infected with bloodstream forms expressing *TbAEK1* gatekeeper mutants (M138G) delayed parasitemia and death, with some mice being apparently cured of infection. Finally, the L. mexicana *AEK1* ortholog has been also considered likely essential in promastigotes because no null mutants could be generated in a kinome-wide gene deletion library (20).

*TcAEK1* null mutants could not be generated carrying out a methodology that has been previously used successfully for obtaining several gene KO cell lines (32). Instead, we obtained single allele KO parasites, which gives support to the essentiality of this gene for T. cruzi. In addition, our results also suggest that gene or chromosome duplication could have arisen when a second allele was targeted for disruption. Retention of essential genes following attempts at generating gene deletion cell lines is a common mechanism observed in *Leishmania* and is related to drug resistance, gene expression regulation, and adaptation to the host environment (20, 62–64). Variation in DNA copy number associated with drug selection and resistance has also been described for T. cruzi (6, 65).

The TcAEK1 amino acid sequence includes several features of the AGC kinase group of protein kinases, although the domain architecture outside the catalytic region is less conserved. In addition, no apparent *AEK1* orthologs can be found in mammals. Many AGC kinases are activated by serine/threonine phosphorylation of two highly conserved motifs by the 3-phosphoinositide-dependent kinase-1 (PDK-1): the activation loop in the catalytic domain and the hydrophobic motif, C-terminal to the kinase domain (66). Phosphoproteomic analyses in T. brucei and T. cruzi indicate that phosphoserines are present at these two domains in AEK1 orthologs of both species (10, 14, 67). Additionally, a PDK-1 homolog is present in the T. cruzi genome (15, 17) (TriTrypDB ID: TcYC6_0028570 for T. cruzi Y C6 strain). Although functional evidence is absent, the data suggest a conserved activation/modulation mechanism for members of the AGC group of protein kinases in T. cruzi and for TcAEK1 in particular.

Interestingly, although tubulin may not be an optimal housekeeping protein for normalization when comparing protein expression levels between different T. cruzi developmental stages, our results suggest that the expression level of the endogenously tagged TcAEK1 protein was higher in the replicative stages (epimastigotes and amastigotes) than in the nondividing trypomastigote forms. The *TcAEK1*-SKO epimastigotes exhibited an increased *in vitro* metacyclogenesis, which correlates with the significant number of metacyclic trypomastigotes observed in mutant epimastigotes in the exponential growth phase in LIT medium. A potential explanation may be that the downregulation of *TcAEK1* could lead epimastigotes to “escape” into a nonreplicative form. Similarly, T. cruzi epimastigotes exhibited an increased metacyclogenesis along with impairment of cytokinesis when they were cultured with an inhibitor of casein kinase 2 (61).

The defects observed in different T. cruzi developmental stages after knocking down or inhibiting TcAEK1 with BKIs suggest that this protein kinase is a pleiotropic player involved in crucial cellular processes in T. cruzi. In the absence of efficient inducible downregulation methods in T. cruzi, and lacking specific inhibitors, our work highlights the relevance of generating ATP analog-sensitive mutants in T. cruzi by CRISPR/Cas9-based strategies for functional studies of protein kinases and validation of therapeutic targets. Moreover, the use of selective and/or specific inhibitors permits an almost instantaneous loss of activity in a population of mutant cells, preventing a gradual reduction of proteins and adaptation of mutant cells.

In summary, by analyzing single allele deletion and engineered ATP analog-sensitive cell lines obtained using CRISPR/Cas9 technology, we demonstrated that *TcAEK1* is critical for successful transit through multiple life cycle stages and the generation of pathogenic stages. It is essential for epimastigote proliferation, trypomastigote host cell invasion, and amastigote replication. We also demonstrated its requirement for cytokinesis in the epimastigote form. The use of BKIs against ATP analog-sensitive *TcAEK1* allows us to consider this protein as a validated drug target, providing further impetus to develop and/or test inhibitor molecules *in vitro* and *in vivo.*

## MATERIALS AND METHODS

### Chemicals and reagents.

MitoTracker Deep Red FM, Alexa-conjugated secondary antibodies, Pierce ECL Western blotting substrate, and BCA protein assay kit were from Thermo Fisher Scientific Inc. Blasticidin S HCl was from AG Scientific (San Diego, CA). BLUelf prestained protein ladder was from FroggaBio (Wheatfield, NY). Puromycin was from Acros Organics (Fair Lawn, NJ). Anti-HA.11 epitope tag monoclonal antibody was from BioLegend (San Diego, CA). Alexa Fluor-conjugated secondary antibodies and horseradish peroxidase (HRP)-conjugated secondary antibodies were purchased from Life Technologies. Benzonase nuclease was from Novagen (EMD Millipore, Billerica, MA). GoTaq DNA polymerase and T4 DNA ligase were from Promega (Madison, WI). Antarctic phosphatase, restriction enzymes, and Q5 high-fidelity DNA polymerase were from New England Biolabs (Ipswich, MA). Fluoromount-G was from SouthernBiotech (Birmingham, AL). Nitrocellulose membrane and alamarBlue were from Bio-Rad (Hercules, CA). DNA oligonucleotides were purchased from Integrated DNA Technologies, Inc. (Coraville, IA.). Escherichia coli DH5α Mix & Go competent cells, ZymoPURE plasmid midiprep kit, and ZymoPURE plasmid miniprep kit were from Zymo Research (Irvine, CA). G418 sulfate was from KSE Scientific (Durham, NC). Anti-tubulin monoclonal antibody, anti-Flag antibody, mammalian cell protease inhibitor mixture (Sigma P8340), other protease inhibitors, and all other reagents of analytical grade were from Sigma (St. Louis, MO). Rabbit polyclonal antibody against cruzipain was a gift from Vanina Alvarez (Universidad Nacional de San Martin, Argentina). The pMOTag23M vector (68) was from Thomas Seebeck (University of Bern, Bern, Switzerland). Polyclonal antibody against TbBIP was from Jay Bangs (State University of New, York, Buffalo, NY). Stock solutions of bumped kinase inhibitors (BKI) 1294 (38) and 1553 (39) (20 mM) were kindly provided by Dustin Maly and Rama S.R. Vidadala (University of Washington).

### Cell cultures.

T. cruzi Y strain epimastigotes were cultured in liver infusion tryptose (LIT) medium containing 10% heat-inactivated fetal bovine serum (FBS) at 28°C (69). CRISPR/Cas9 mutant cell lines were maintained in medium containing 250 μg/ml G418 and 10 μg/ml blasticidin or 5 μg/ml puromycin. Parasites from the control cell lines transfected with scrambled sgRNA (scrambled) or pTREX-n empty vector (EV) and overexpressing *TcAEK1-*sgRNA-787/Cas9, *TcAEK1*-3×HA, or *TcAEK1*-Flag were cultured in medium containing 250 g/ml G418. The growth rate of epimastigotes was determined by counting cells in a Neubauer chamber. Tissue culture cell-derived trypomastigotes were obtained from Vero cells infected with metacyclic trypomastigotes obtained as described below. T. cruzi trypomastigotes were collected from the culture medium of infected host cells as described previously (70). Vero cells were grown in RPMI supplemented with 10% fetal bovine serum and maintained at 37°C with 5% CO_2_.

### *In silico* analysis.

Prediction of protein domains of TcAEK1 amino acid sequence was made using web servers https://www.ebi.ac.uk/interpro/ and https://prosite.expasy.org/scanprosite/. WoLF PSORT method (https://wolfpsort.hgc.jp/) was used to predict subcellular localization sites based on amino acid sequences. For *in silico* analyses for selection of protospacers for knockout and tagging of *TcAEK1* gene, we used the CRISPR/Cas9 genome editing method for T. cruzi developed before (29, 71, 72). Selection of protospacers for both strategies was performed using EuPaGDT (eukaryotic pathogen CRISPR guide RNA/DNA design tool; http://grna.ctegd.uga.edu) (73).

### *TcAEK1* overexpression.

*TcAEK1* open reading frame (ORF) (1,179 nt) was PCR amplified using T. cruzi Y strain genomic DNA as the template (primers 1 and 2; Table S1) and cloned into the pTREX-n/3×HA vector (31) by restriction sites XbaI/XhoI. Gene cloning was confirmed by PCR and sequencing, and constructs were subsequently used to transfect T. cruzi epimastigotes. *TcAEK1* overexpression was confirmed by Western blotting using anti-HA antibodies.

### *TcAEK1* endogenous C-terminal tagging and mutation.

CRISPR/Cas9-mediated endogenous C-terminal tagging of TcAEK1 was done as described previously (71, 72). We chose a specific sgRNA sequence targeting the 3′ end of *TcAEK1* gene (TriTrypDB ID: TcYC6_ 0120630). The construct *TcAEK1-*Ctag-sgRNA/Cas9/pTREX-n (primers 3 and 4; Table S1), together with a DNA donor cassette to induce homology-directed repair, was used to cotransfect T. cruzi epimastigotes and to insert a specific 3xc-Myc-tag sequence at the 3′ end of the gene. DNA donor cassette containing the 3xc-Myc-tag sequence and the puromycin resistance gene was amplified using the pMOTag23M vector (68) as the template (primers 5 and 7; Table S1). One additional DNA donor cassette was designed to generate amino acid substitutions, along with the insertion of the 3xc-Myc-tag, from HLNK to GLNG amino acid residues at the C terminus (residues 388 to 391) of TcAEK1 (primers 6 and 7; Table S1). Epimastigotes cotransfected with *TcAEK1-*Ctag-sgRNA/Cas9/pTREX-n plasmid and any of the DNA donor cassettes were cultured for 5 weeks with G418 and puromycin for selection of double-resistant parasites. Endogenous gene tagging was verified by PCR from gDNA using primers 8 and 9 (Table S1) and by Western blotting.

### CRISPR-Cas9 mediated deletion of a single allele of *TcAEK1*.

We used the *TcAEK1* sequence from T. cruzi Y C6 strain to design a single guide RNA (sgRNA), which is predicted to target DNA cleavage at nucleotide +787 of *TcAEK1* (*TcAEK1-*sgRNA-787)*. TcAEK1-*sgRNA-787 was PCR amplified (primers 4 and 10; Table S1) and cloned into Cas9/pTREX-n as described previously (29, 30) to generate *TcAEK1-*sgRNA-787/Cas9/pTREX-n plasmid. A scrambled sgRNA cloned in Cas9/pTREX-n was used as the control. In the three strategies (see Fig. 3A, Fig. S2A, and Fig. S3A) designed to try to generate a *TcAEK1-*KO cell line, 3 different linear DNA donor cassettes were obtained by PCR using a set of 120-nt primers (ultramers). In the first and second strategies, each one of the ultramers had 20 nt annealing on the blasticidin S-deaminase (*Bsd*) gene (primers 11, 12, and 14; Table S1), whereas for the third strategy, ultramers allowed the amplification of the puromycin N-acetyltransferase gene (*Pac*) (primers 13 and 15; Table S1). We first transfected epimastigotes with *TcAEK1-*sgRNA-787/Cas9/pTREX-n plasmid, and after obtaining a homogeneous G418-resistant population expressing Cas9-GFP in the nuclei of epimastigotes, the *TcAEK1-*sgRNA-787/Cas9 cells were transfected with a repair cassette fragment containing the *Bsd* resistance marker to promote the disruption of *TcAEK1* ORF. Transfected cells were selected with G418 and blasticidin. For the third strategy, *TcAEK1-*sgRNA-787/Cas9 cells with one allele disrupted by *Bsd* cassette (*TcAEK1*-SKO) were transfected with a DNA donor cassette fragment containing the *Pac* resistance marker to promote the disruption of the WT remaining *TcAEK1* allele. Stable transfected cells were obtained by selection with G418, blasticidin, and puromycin. *TcAEK1* gene disruption was verified by PCR using primers 9 and 16 (Table S1).

### Generation of ATP analog-sensitive *TcAEK1*.

To generate parasite cell lines with *TcAEK1* mutated at the gatekeeper residue, we followed a CRISPR/Cas9-induced knock-in strategy based on that we used previously (40). The design provides a DNA donor molecule to induce homologous-directed repair, which introduces mutations at the gatekeeper residue M125 (to generate substitution to alanine or glycine) and silent mutations in the protospacer sequence to prevent the constitutively expressed sgRNA and Cas9 from continuing to cleave their targets. In addition, the repair cassettes also allowed tagging of the mutated gene to follow its expression by immunofluorescence analysis and Western blotting. We chose sgRNA-386 that directs DNA cleavage near codon 125 of *TcAEK1* (primer 17; Table S1).

To generate donor DNA constructs, we performed a two-step-PCR strategy. In the first step, we amplified in two separate PCRs a 5′ portion of the gene including a 120-nt ultramer with the WT or mutated *TcAEK1* gatekeeper residue M125 (primers 18 to 21; Table S1), while in the second step, the 3xc-Myc-tag sequence and the puromycin resistance gene were amplified using the pMOTag23M vector as the template (primers 7 and 22; Table S1) (68). Subsequently, we performed an overlap extension PCR including the two above-mentioned fragments as the templates (primers 7 and 18 to 20; Table S1). Finally, the resulting PCR amplicons were used as donor DNAs to generate these mutant cells: *TcAEK1*^WT^, *TcAEK1*^M125A^, and *TcAEK1*^M125G^. Epimastigotes cotransfected with *TcAEK1*-sgRNA-386/Cas9/pTREX-n and DNA donor were cultured for 5 weeks with G418 and puromycin for selection of double-resistant parasites and then cloned by limiting dilution. Endogenous gene tagging was verified by PCR from gDNA using specific primer sets (primers 8 and 9; Table S1) and by Western blotting. Mutation at the *TcAKE1* gatekeeper residue was confirmed by amplifying gDNA with primers 9 and 23 (Table S1). Electrophoresed DNA fragments were gel-purified and submitted to determine their nucleotide sequence using primer 24 (Table S1).

### Cell transfections.

T. cruzi Y strain epimastigotes were transfected as described previously (31). Briefly, T. cruzi epimastigotes in early exponential phase (4 × 10^7^ cells) were washed with phosphate-buffered saline (PBS; pH 7.4) at room temperature (RT) and transfected in ice-cold CytoMix (120 mM KCl, 0.15 mM CaCl_2_, 10 mM K_2_HPO_4_, 25 mM HEPES, 2 mM EDTA, 5 mM MgCl_2_ [pH 7.6]) containing 25 μg of each plasmid construct and 25 μg of donor DNA in 4-mm electroporation cuvettes with three pulses (1,500 V, 25 microfarads) delivered by a Gene Pulser Xcell electroporation system (Bio-Rad). Transfected epimastigotes were cultured in LIT medium supplemented with 20% heat-inactivated FBS until stable cell lines were obtained. When needed, the antibiotic used for drug selection and maintenance was 250 μg/ml G418, 10 μg/ml blasticidin, or 5 μg/ml puromycin. Parasite clones were obtained by limiting dilution.

### Western blotting.

Western blotting was carried out as described previously (74). Briefly, parasites were harvested and washed twice in PBS and subsequently resuspended in radio-immunoprecipitation assay buffer (RIPA: 150 mM NaCl, 20 mM Tris-HCl [pH 7.5], 1 mM EDTA, 1% SDS, 0.1% Triton X-100) plus a mammalian cell protease inhibitor mixture (diluted 1:250), 1 mM phenylmethylsulfonyl fluoride, 2.5 mM tosyl phenylalanyl chloromethyl ketone (TPCK), 100 M *N*-(*trans*-epoxysuccinyl)-l-leucine 4-guanidinobutylamide (E64), and benzonase nuclease (25 units/ml of culture). The cells were then incubated for 1 h on ice and protein concentration was determined by bicinchoninic acid (BCA) protein assay. Thirty micrograms of protein from each cell lysate was mixed with Laemmli sample buffer (125 mM Tris-HCl [pH 7], 10% [wt/vol] β-mercaptoethanol, 20% [vol/vol] glycerol, 4.0% [wt/vol] SDS, 4.0% [wt/vol] bromphenol blue) before application to 10% SDS-polyacrylamide gels. Electrophoresed proteins were transferred onto nitrocellulose membranes with a Bio-Rad transblot apparatus. Membranes were blocked with 5% nonfat dried skim milk in PBS-T (PBS containing 0.1% vol/vol Tween 20) overnight at 4°C. Next, blots were incubated for 1 h at RT, with a primary antibody, i.e., monoclonal anti-HA (1:5,000), monoclonal anti-c-Myc-tag (1:100), rabbit anti-Flag (1:20,000), and monoclonal anti-tubulin (1:40,000). After three washes with PBS-T, blots were incubated with the secondary antibody (goat anti-mouse IgG or goat anti-rabbit IgG, HRP-conjugated antibody, diluted [1:10,000]). Membranes were washed three times with PBS-T, and then blots were incubated with Pierce ECL plus substrate and images were obtained and processed with a ChemiDoc Imaging System (Bio-Rad). Blot densitometry analyses were performed with ImageJ v1.48 software.

### Immunofluorescence analysis.

Cells were washed with PBS and fixed with 4% paraformaldehyde in PBS for 1 h at RT. Cells were allowed to adhere to poly-l-lysine-coated coverslips and then permeabilized for 5 min with 0.1% Triton X-100. Permeabilized cells were blocked with PBS containing 3% bovine serum albumin (BSA), 1% fish gelatin, 50 mM NH_4_Cl, and 5% goat serum overnight at 4°C. Then, cells were incubated with a primary antibody (monoclonal anti-HA-Tag [1:50], monoclonal anti-c-Myc-tag [1:10], monoclonal anti-TcFCaBP [1:10], rabbit polyclonal anti-TcBIP [1:50], or rabbit polyclonal anti-cruzipain [1:100]) diluted in 1% BSA in PBS (pH 8.0) for 1 h at RT. Cells were washed three times with 1% BSA in PBS (pH 8.0) and then incubated for 1 h at RT in the dark with Alexa Fluor 488- or Alexa Fluor 546-conjugated goat anti-mouse secondary antibodies (1:1,000). Then, cells were washed and mounted on slides using Fluoromount-G mounting medium containing 5 μg/ml of 4,6-diamidino-2-phenylindole (DAPI) to stain DNA. Controls were done as described above but in the absence of a primary antibody. Differential interference contrast and fluorescence optical images were captured with a 100× objective (1.35-aperture) lens under nonsaturating conditions with an Olympus IX-71 inverted fluorescence microscope with a Photometrix CoolSnapHQ charge-coupled device camera driven by DeltaVision software (Applied Precision, Issaquah, WA); images were then deconvolved for 15 cycles using Sotwarx deconvolution software.

### Metacyclogenesis.

Metacyclic trypomastigotes were obtained following the protocol described by Bourguignon et al. (75) with minor modifications. Epimastigotes were obtained after 4 days in LIT medium and incubated for 2 h in triatome artificial urine (TAU) medium (190 mM NaCl, 17 mM KCl, 2 mM MgCl_2_, 2 mM CaCl_2_, 0.035% sodium bicarbonate, 8 mM phosphate [pH 6.9]) at room temperature. Then, parasites were incubated for 96 h in TAU 3AAG medium (TAU medium supplemented with 10 mM l-proline, 50 mM sodium l-glutamate, 2 mM sodium l-aspartate, and 10 mM glucose). Samples were harvested from the TAU 3AAG plus FBS-containing medium at day 4 of cultivation, washed with PBS, and fixed with 4% paraformaldehyde in PBS for 1 h at RT. Cells were adhered to poly-l-lysine-coated coverslips and mounted on slides using Fluoromount-G mounting medium containing 5 μg/ml of DAPI to stain DNA. For incubation with drugs, the metacyclic trypomastigotes were purified using anion-exchange chromatography in DEAE cellulose columns as described previously (76).

### Invasion and replication assays.

Gamma-irradiated (2,000 radiation-absorbed doses) Vero cells (4.5 × 10^5^ cells) were plated onto sterile coverslips in a 12-well plate and incubated overnight at 37°C with 7% CO_2_ in RPMI medium supplemented with 10% fresh FBS. Tissue culture-derived trypomastigote collections were incubated at 4°C overnight to allow amastigotes to settle from swimming trypomastigotes. Trypomastigotes from the supernatants of these collections were counted and used to infect the mammalian cells at a 10:1 ratio of parasites to host cells. At 4 h postinfection, coverslips were washed extensively with Hanks’ balanced salt solution, followed by PBS (pH 7.4), to remove any extracellular parasites. This protocol has been optimized with a multiplicity of infection (MOI) of 10 to ensure that at most one trypomastigote (Y strain) invades each irradiated host cell. Coverslips were immediately fixed in 4% paraformaldehyde in PBS (pH 7.4) at 4°C for 30 min and then washed once with PBS and counterstained with DAPI (15 μg/ml) in Fluoromount-G mounting medium on glass slides, which stains host and parasite DNA. Coverslips were analyzed on an Olympus BX60 microscope to quantify the number of host cells that contained intracellular parasites and the number of intracellular parasites per cell in 40 randomly selected fields. Four hundred host cells were counted per sample in three independent experiments. To quantify amastigote replication, Vero cells were infected at a 10:1 ratio of parasites to host cells, and after they were washed at 4 h postinfection as described above, coverslips were allowed to incubate for 48 h at 35°C with 7% CO_2_ prior to fixation and DAPI staining.

### Drug solutions.

Stock solutions of bumped kinase inhibitors (BKIs) 1294 (38) and 1553 (39) (20 mM) were prepared in dimethyl sulfoxide (DMSO), with the final concentration of DMSO in the experiments never being higher than 0.2%.

### Effects of BKIs in ATP analog-sensitive *TcAEK1* mutants.

To calculate the concentration of BKI-1294 and BKI-1553 that inhibited 50% epimastigote growth (IC_50_), 100-μl log phase parasites (2.5 × 10^6^/ml) were incubated at 28°C with different concentrations of drugs (0.01 to 20 μM) in biological triplicates for 48 h. After 48 h, 20 μl of alamarBlue (Bio-Rad) was added to each plate, allowing color changes in response to the chemical reduction by viable trypanosomes. Plates were incubated for an additional 8 h. Later, fluorescence was quantified at an excitation wavelength of 570 nm and an emission wavelength of 590 nm using a microplate reader (BioTek Instruments, Winooski, VT) driven by Gen5 software for data collection and analysis. To calculate the IC_50_ for metacyclic trypomastigotes, the parasites (2.5 × 10^6^ cells/ml) were incubated with different concentrations of BKI-1294 and BKI-1553 (0.1 to 40 μM) in biological triplicates. After 24 h at 28°C, the parasite number was counted in a Neubauer chamber. In both epimastigotes and metacyclic trypomastigotes, the death percentage was estimated relative to the untreated control (LIT medium with 0.1% DMSO), generating dose-effect curves. GraphPad Prism software was used to calculate the IC_50_ values employing the death percentage for each BKI concentration. Each experiment was performed on three independent occasions.

### Flow cytometry analysis.

For flow cytometry analysis, 1 × 10^6^ cells were harvested by centrifugation, washed with PBS, and fixed in ice-cold 70% ethanol/30% PBS for 30 min. Then, they were washed once with PBS and resuspended in 1 ml of staining solution (20 μg/ml propidium iodide, 38 mM citrate buffer [pH 7.40], 20 μg/ml RNase, 0.3% NP-40). After the sample was inverted three times, the cell suspension was incubated at 37°C for 30 min. DNA content of propidium iodide-stained cells was analyzed using a CyAn ADP analyzer flow cytometer (Beckman Coulter, Hialeah, FL) at the CTEGD Cytometry Shared Resource Lab (UGA).

### Scanning and transmission electron microscopy.

Cells were fixed in 2.5% glutaraldehyde in 0.1 M cacodylate-HCl buffer (pH 7.25) and subsequently processed for transmission electron microscopy (TEM) and scanning electron microscopy (SEM) at the Electron Microscopy Facility of the Department of Pathology, College of Veterinary Medicine, University of Georgia, Athens, GA. TEM images were obtained with a JEOL JEM1011 transmission electron microscope (JEOL Tokyo, Japan) equipped with a high-contrast 2k by 2k AMT midmount digital camera from Advanced Microscopy Techniques, Corp. (Woburn, MA). Parasites for SEM were viewed with a Teneo field-emission scanning electron microscope (ThermoFisher, Hillsboro, OR).

### Statistical analysis.

Statistical analyses were performed with GraphPad Prism software (La Jolla, CA), version 6.0. Reported values are means ± standard deviation (SD) of *n* independent experiments, as indicated in the figure legends. The level of significance was evaluated by Student’s *t* test for comparisons between two cell lines, one-way analysis of variance (ANOVA) for comparisons between more than two cell lines, and two-way ANOVA with multiple-comparison tests for analyses of grouped data.
