# Supplementary material for: Drug Target Validation of the Protein Kinase AEK1, Essential for Proliferation, Host Cell Invasion, and Intracellular Replication of the Human Pathogen Trypanosoma cruzi
Source: Microbiol Spectr. 2021 Sep 29;9(2):e00738-21. doi: 10.1128/Spectrum.00738-21 (PMC8557885; doi:10.1128/Spectrum.00738-21)
Supplement: SUPPLEMENTAL FILE 1 — Supplemental material. Download SPECTRUM00738-21_Supp_1_seq11.pdf, PDF file, 7.3 MB [file spectrum00738-21_supp_1_seq11.pdf]

Supplementary Information

Drug Target Validation of the Protein Kinase *AEK1,* Essential for Proliferation, Host Cell Invasion, and Intracellular Replication of the Human Pathogen *Trypanosoma cruzi*

Miguel A. Chiurillo[1,2,*], Bryan C. Jensen[3], and Roberto Docampo[1,*]

[1]Center for Tropical and Emerging Global Diseases, and Department of Cell Biology, University of Georgia, Athens, GA, 30602, USA

[2]Department of Biological Sciences, University of Cincinnati, Cincinnati, OH 45221

[3]Seattle Children's Research Institute, Seattle, WA 98109

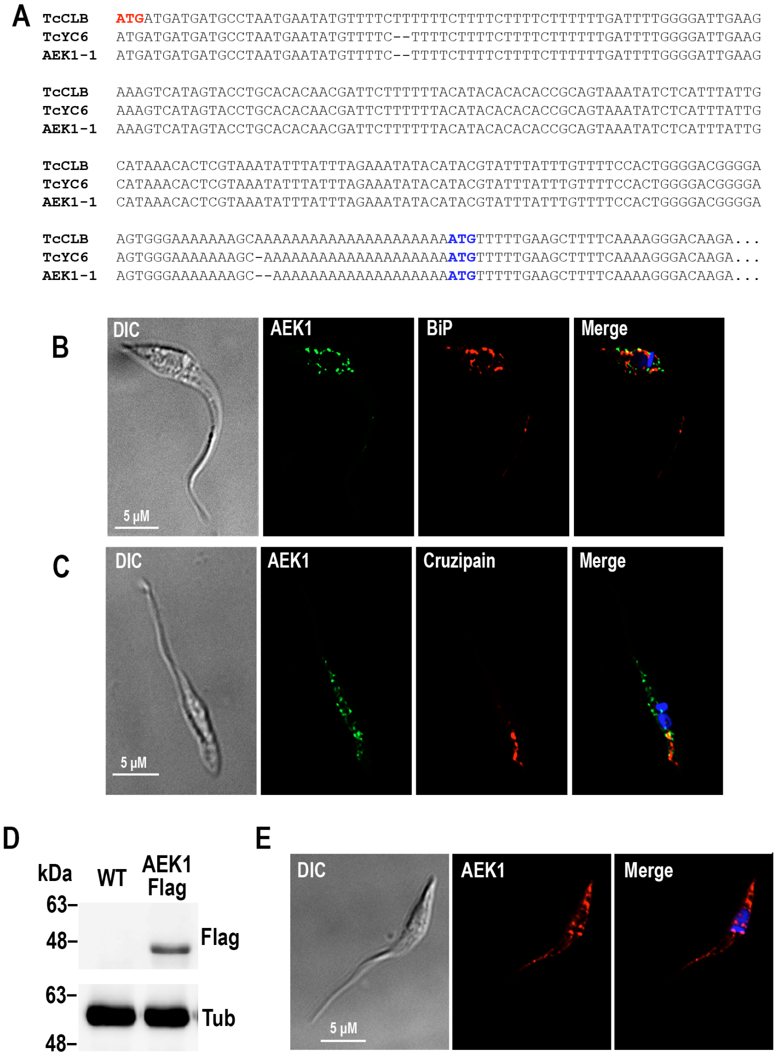

FIG S1 TcAEK1 subcellular localization. **(A)** Nucleotide sequence alignment the 5' end of annotated *TcAEK1 T. cruzi* CL Brener strain (Non-Esmeraldo like haplotype, TriTrypDB ID: TcCLB.508479.150) with the 5' UTRs sequence of *TcAEK1* from *T. cruzi* YC6 strain (TriTrypDB ID: TcYC6_012063), and PCR amplified in this work from *T. cruzi* Y strain genomic DNA (AEK1-1). The annotated start codon for the TcCLB ortholog is indicated in *red*. However, nucleotide differences in this region, in addition to the estimated molecular weight of the overexpressed *TcAEK1* protein in *T. cruzi* Y strain, indicates that the true ATG start codon, at least in this strain, is that shown in *blue*. (B and C) Fluorescence microscopy of endogenously tagged *TcAEK1*-3xc-Myc epimastigotes indicates very little co-localization with ER (B, BIP) and reservosome (C, Cruzipain) markers. TcAEK1-3xc-Myc was detected with monoclonal anti-c-Myc antibody (*green*). Polyclonal antibodies anti-TbBiP (B) and anti-TcCruzipain (C) were used to label ER and reservosomes, respectively (*red*). The *merge* shows co-localization signals in *yellow*. DIC images are shown in the left panel. Nucleus and kinetoplast were labeled with DAPI (*blue*). Scale bars, 5 µm. (D) An internal Flag tag (DYKDDDDK) was inserted into the N-terminal coding sequence of *TcAEK1*. Subsequently the tagged gene (*TcAEK1*-Flag) was cloned into pTREX-n vector and used to transfect *T. cruzi* epimastigotes. *TcAEK1*-Flag (AEK1-Flag) overexpression was confirmed by western blot analysis using anti-Flag antibodies. Wild type cells (WT) were used as control cell line. Tubulin (Tub) was used as loading control. (E) IFA showed subcellular localization of overexpressed TcAEK1-Flag detected with anti-Flag antibodies (AEK1-Flag [*red*]) in *T. cruzi* epimastigotes. Merge of red signal, DAPI staining (*blue*), and DIC (differential interference contrast) images is also shown. Scale bar = 5 µm.

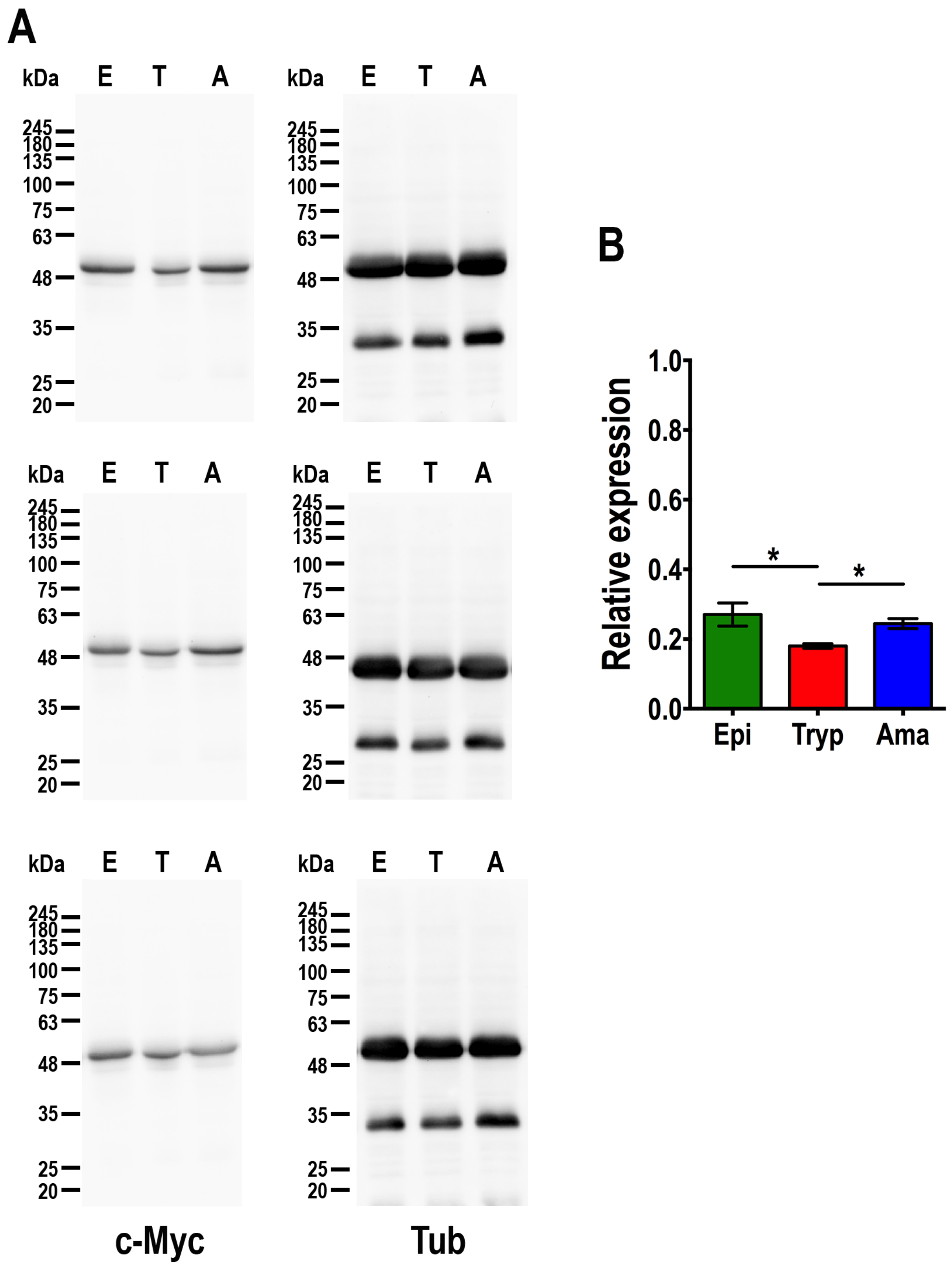

FIG S2 Expression of endogenously tagged *TcAEK1*-3xc-Myc in the three life cycle stages of *T. cruzi*. (A) Complete western blots (3 experiments) of *TcEK1*-3xc-Myc in total extracts of epimastigotes (E), tissue culture cell-derived trypomastigotes (T), and amastigotes (A) using anti-c-Myc antibodies. Tubulin was used as loading control. (B) Densitometry analysis of three western blots. Values are means ± S.D. (n = 3). *P < 0.05 (one-way ANOVA with Tukey's multiple comparisons test).

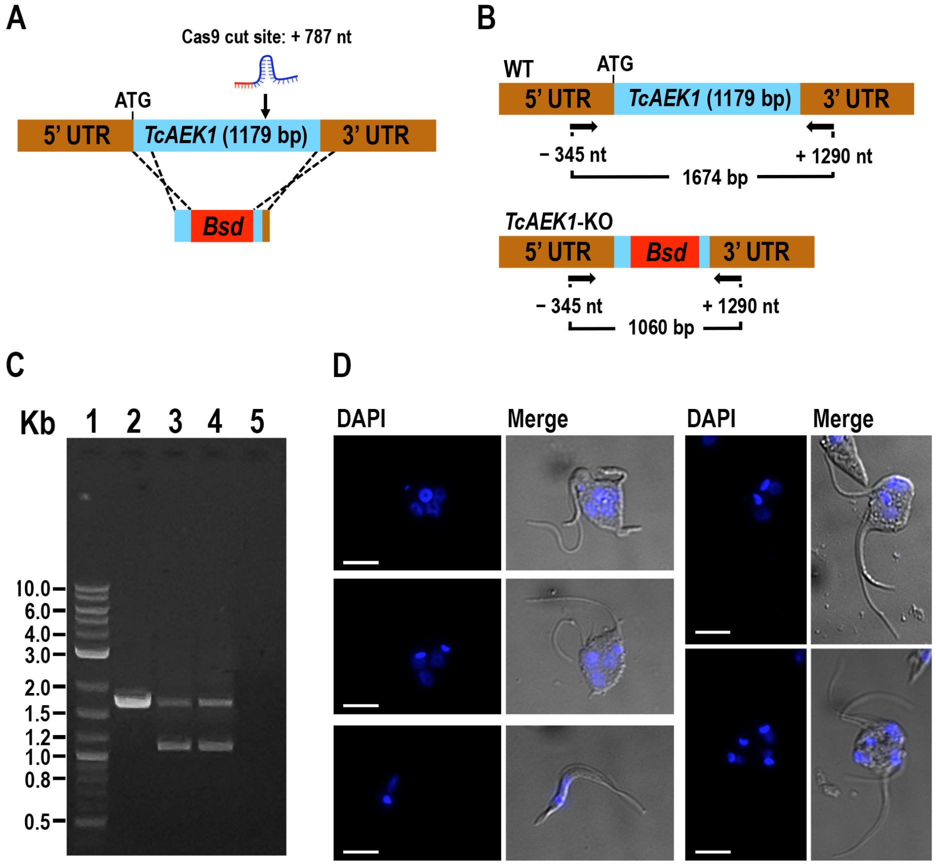

FIG S3 Generation of *TcAEK1*-SKO cells by a second strategy. (A) Schematic representation of the second strategy (using a different DNA donor) designed to generate a *TcAEK1*-KO mutant by CRISPR/Cas9-induced homologous recombination. Epimastigotes constitutively expressing Cas9 and sgRNA that can originate a DNA double-stranded break at nt +787 of the *TcAEK1* ORF (1,179 bp) were transfected with a blasticidin *S*-deaminase (*Bsd*) cassette containing ~100-bp homologous regions spanning from nt +1 to +96 and from nt +1094 to +1190 of the *TcAEK1* locus to induce homologous -directed repair. (B) PCR primers used to verify *TcAEK1* ablation. Arrows indicate primers added to the reaction. The intact locus generates a PCR product of 1,674 bp, while the disrupted locus generates a DNA fragment of 1,060 bp. UTR, untranslated region. (C) Only one *TcAEK1* allele was disrupted at its genomic locus in the SKO cell lines. Lanes: 1, 1-kb plus ladder; 2, WT; 3, *TcAEK1*-SKO-2 population #1; 4, *TcAEK1*-SKO-2 population #2; 5, PCR negative control. (D) Representative images displaying aberrant cell morphology and DNA content of *TcAEK1*-SKO-2 epimastigotes, as well as trypomastigote cell originated in exponential culture in LIT medium (lower left panel). DAPI staining (*blue*) and merge with DIC images are shown. Scale bars = 5 µm.

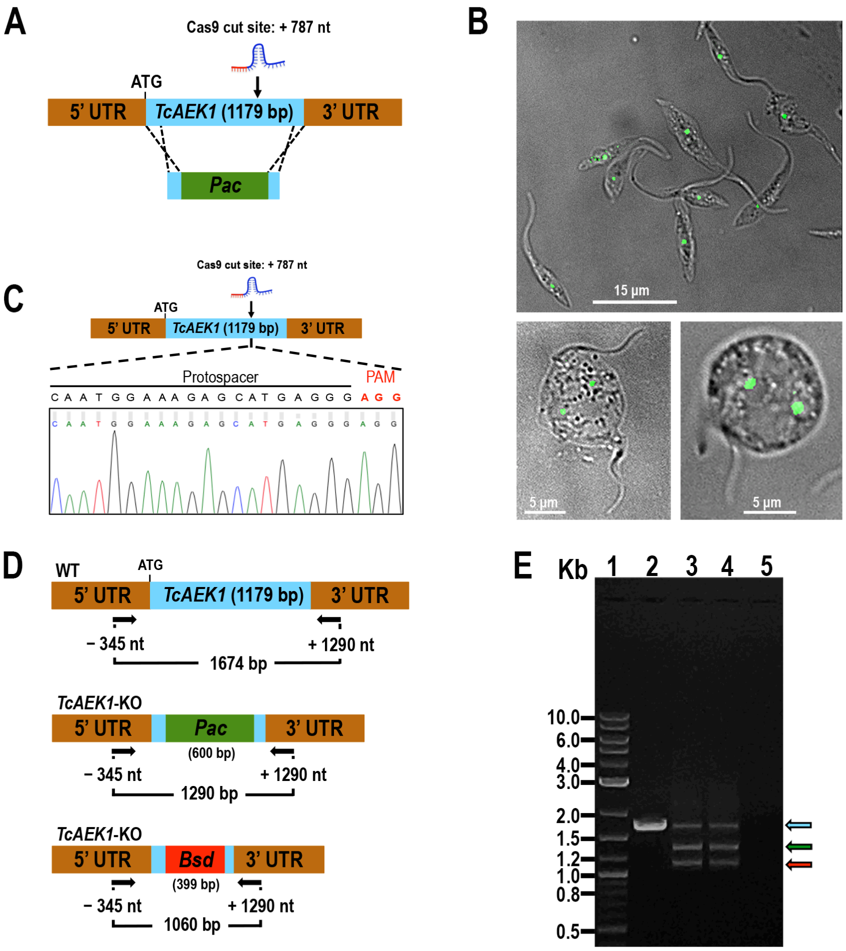

FIG S4 Generation of *TcAEK1*-SKO cells by a third strategy. A third strategy was designed to try to generate a *TcAEK1* KO cell line, *TcAEK1*-SKO-2 epimastigotes were transfected with a puromycin N-acetyltransferase cassette (*Pac*). (A) Schematic representation of the third strategy designed to generate a *TcAEK1*-KO mutant by CRISPR/Cas9-induced homologous recombination. *TcAEK1*-SKO-2 epimastigotes constitutively expressing Cas9 and sgRNA that originate a DNA double-stranded break at nt +787 of the *TcAEK1* ORF were transfected with a *Pac* cassette containing ~100-bp homologous regions spanning from nt +1 to +96 and from nt +1094 to +1190 of the *TcAEK1* locus to induce homologous directed repair. (B) Constitutive nuclear expression of fluorescent Cas9-GFP was confirmed in *TcAEK1*-SKO-2 epimastigotes and multinucleated cells by fluorescence microscopy. Panels show merged images of DIC and green (GFP) fluorescence. (C) Integrity of the protospacer nucleotide sequence for sgRNA-787 in *TcAEK1*-SKO-2 cells was confirmed by checking the DNA sequence of the amplified *TcAEK1* WT allele. (D) PCR primers used to verify *TcAEK1* ablation. Arrows indicate primers added to the reaction. The intact locus generates a PCR product of 1,674 bp, while the loci disrupted with *Pac* and *Bsd* cassettes generate DNA fragments of 1,290 bp and 1,060 bp, respectively. UTR, untranslated region. (E) Two disrupted *TcAEK1* alleles at their genomic loci and one WT was detected by PCR in the SKO-3 cell lines (blasticidin/puromycin resistant). Lanes: 1, 1-kb plus ladder; 2, WT; 3, *TcAEK1*-SKO-3 population #1; 4, *TcAEK1*-SKO-3 population #2; 5, PCR negative control. Color of arrows (*right*) indicates the three amplified fragments corresponding to WT *TcAEK1* (light blue), and *Pac* (green) and *Bsd* (red) containing DNA fragments as shown in (D).

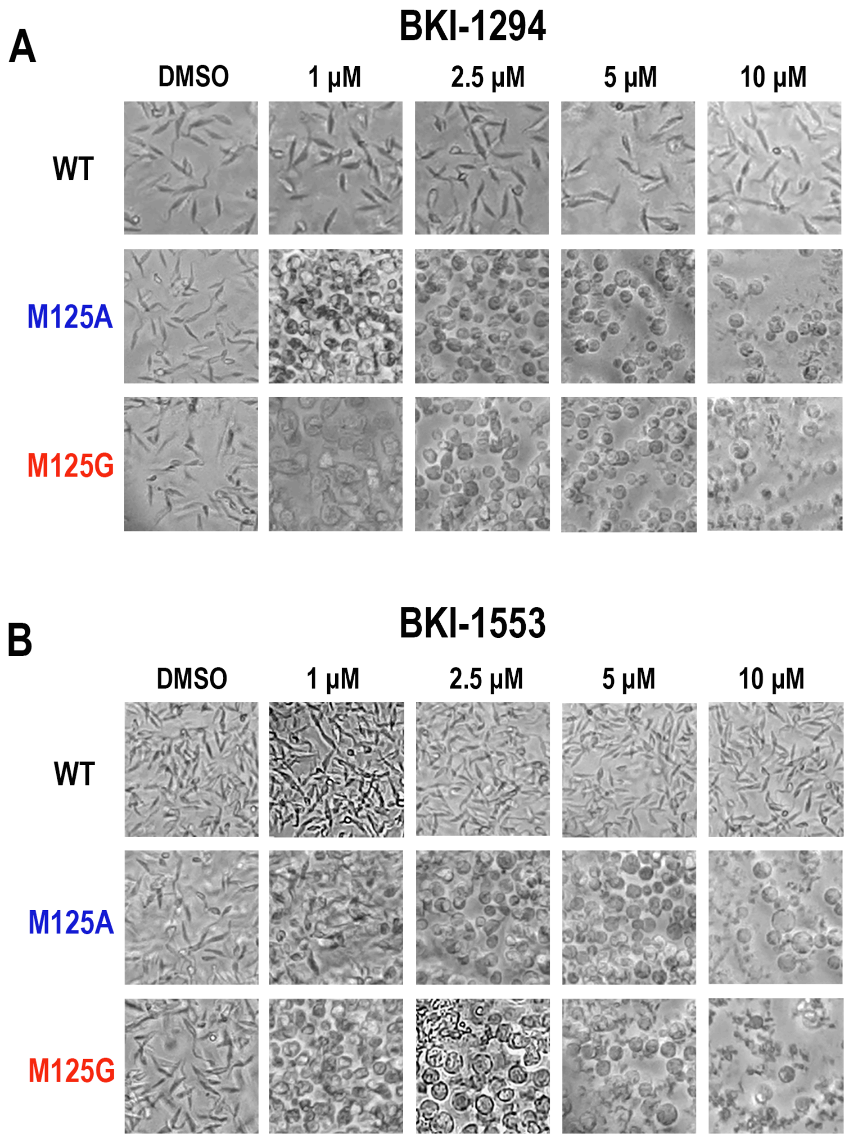

FIG S5 BKIs and effects on *TcAEK1* gatekeeper mutants. Representative phase images of *TcAEK1*^{WT} (WT), *TcAEK1*^{M125A} (M125A) and *TcAEK1*^{M125G} (M125G) epimastigotes treated with DMSO (0.2%) or 1, 2.5, 5, 10 µM of BKI 1294 (A) or 1553 (B). 2.5 x 10^6 cells (1 ml) in LIT medium supplemented with 10% FBS were incubated for 48h at 27°C.

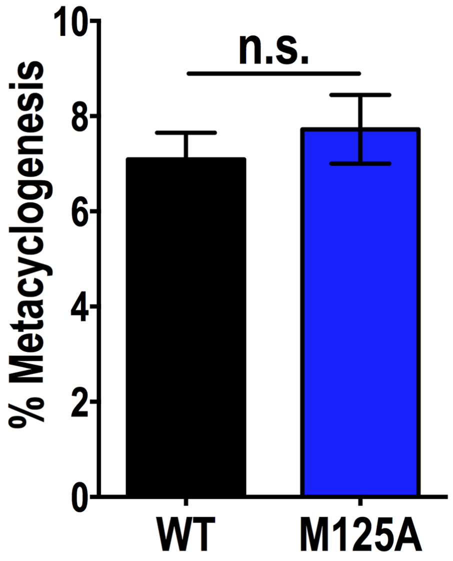

FIG S6 Percentage of metacyclic trypomastigotes in epimastigote culture after incubation in TAU 3AAG medium. *TcAEK1*^{WT} (WT) and *TcAEK1*^{M125A} (M125A) epimastigotes differentiation to metacyclic trypomastigotes was quantified by staining with DAPI to distinguish the position of the kinetoplast by fluorescence microscopy. No significant (n.s.) difference was found using Student's *t* test (*n* = 3).

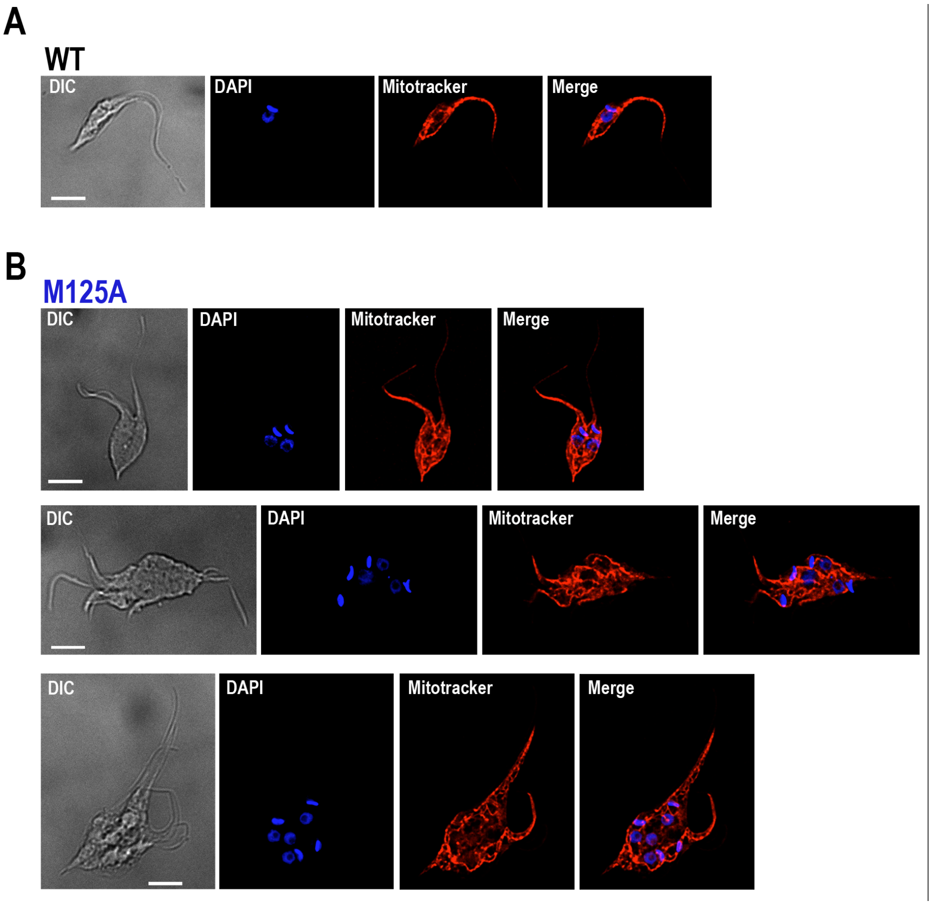

FIG S7 Effect of BKI 1553 on mitochondrial integrity of *TcAEK1* gatekeeper mutants. *T. cruzi* epimastigotes were treated with 1.5 µM of compound 1553 for 48 h. Epimastigotes were then incubated with 100 nM MitoTracker deep red FM for 30 min at 28°C in culture medium before the fixing procedure and visualization by fluorescence microscopy. (A) *TcAEK1*^{WT} (WT), and (B) *TcAEK1*^{M125A} (M125A).

Table S1. Oligonucleotides used in this work.

N°	Primer name	Sequence (5′ → 3′)
1	**FwAEK1-XbaI**	AGTC*TCTAGA*ATGATGATGATGCCTAATGAATATG
2	**RvAEK1-XhoI**	ACTG*CTCGAG*CTAATTTTTATTCAAGTGGTTATCCG
3	**FwAEK1_sgRNA-CTag_BamHI**	GATC*GGATCC***TAACCACTTGAATAAAAATT**GTTTTAGAGCTAGAAATAGC
4	**RvSgRNA**	CAGT*GGATCC*AAAAAAGCACCGACTCGGTG
5	**FwAEK1-CTag-ultra**	**ACTTGTCAATACGCCTGCGCAGTCGTCACAACTGAACTCGCGGCAGCAGCAGCTTTTCACGGGGTTTTCATGCACAGCGGATAACCACTTGAATAAAAAT**GGTACCGGGCCCCCCCTCGAG
6	**FwAEK1_CMut-CTag-ultra**	**ACTTGTCAATACGCCTGCGCAGTCGTCACAACTGAACTCGCGGCAGCAGCAGCTTTTCACGGGGTTTTCATGCACAGCGGATAAC*gg*CTTGAAT*gg*AAAT**GGTACCGGGCCCCCCCTCGAG
7	**RvAEK1-CTAg-ultra**	**TCGCGCCTTCGCCGTCCCCGTGTGTCTCATTTTACACTCCCCTCGCTGGGGAATAAAGAAAAATCAAAAACAAAACAAAACAAACAAAGAAAAAAAAAG**ATGGCGGCCGCTCTAGAACTAGTGGAT
8	**FwAEK1-Ctag-Check**	TGCCAATCCCATCGCCAG
9	**RvAEK1-KO/Ctag-Check**	TTTCTACCCTCGTCGTTCCC
10	**FwAEK1-sgRNA-787_BamHI**	GATC*GGATCC***CAATGGAAAGAGCATGAGGG**GTTTTAGAGCTAGAAATAGC
11	**FwAEK1-KO-ultra-Bsd**	**GCACACAAAGACACAAACGTACCCTCAGATAGAAAGCCCGCGGAATAAAAGGCCTTTGGTGTTTGTGTGTGTGTAGTTTTTCTTGATTTTGTTGTTGTTG**ATGGCCAAGCCTTTGTCTCAAG
12	**FwAEK1-KO-ultra-Bsd2**	**TTGTTTTTGAAGCTTTTCAAAAGGGACAAGAAGGAGAAGGACGAAGAGCGTTCGGGCGAAAAAGCCAGCGAGAAGAAGGTTGGTAACAATAACCATTTGG**ATGGCCAAGCCTTTGTCTCAAG
13	**FwAEK1-KO-ultra-Pac**	**TTGTTTTTGAAGCTTTTCAAAAGGGACAAGAAGGAGAAGGACGAAGAGCGTTCGGGCGAAAAAGCCAG**

		**CGAGAAGAAGGTTGGTAACAATAACCATTTGG**ATGACCGAGTACAAGCCCAC
**14**	**RvAEK1-KO-Ultra-Bsd**	**AAAAAAAAGACCTAATTTTTATTCAAGTGGTTATCCGCTGTGCATGAAAACCCCGTGAAAAGCTGCTGCTGCCGCGAGTTCAGTTGTGACGACTGC**TCATTAGCCCTCCCACACATAACC
**15**	**RvAEK1-KO-Ultra-Pac**	**AAGACCTAATTTTTATTCAAGTGGTTATCCGCTGTGCATGAAAACCCCGTGAAAAGCTGCTGCTGCCGCGAGTTCAGTTGTGACGACTGC**TCATCAGGCACCGGGCTTGCGGG
**16**	**FwAEK1-KO check**	ATGGAGGACATGAACATTGCGG
**17**	**FwAEK1-sgRNA-386_BamHI**	GATC*GGATCC***GTGATGGAATATATGCCAGG**GTTTTAGAGCTAGAAATAGC
**18**	**FwAEK1_gatekeeper_WT**	**AATGTATTGTCACGTATCAATCACCCATATCTTTTGAAGCTTTACTGGACCTTTCAGTCGGAGCATAAGTTGTTTTTTGTGATGGA*g*TA*c*ATGCC*t*GGAGGCGATTTAGACAAATATATGAAC**
**19**	**FwAEK1_gatekeeper_Ala**	**AATGTATTGTCACGTATCAATCACCCATATCTTTTGAAGCTTTACTGGACCTTTCAGTCGGAGCATAAGTTGTTTTTTGT*agc*GGA*g*TA*c*ATGCC*t*GGAGGCGATTTAGACAAATATATGAAC**
**20**	**FwAEK1_gatekeeper_Gly**	**AATGTATTGTCACGTATCAATCACCCATATCTTTTGAAGCTTTACTGGACCTTTCAGTCGGAGCATAAGTTGTTTTTTGT*agg*GGA*g*TA*c*ATGCC*t*GGAGGCGATTTAGACAAATATATGAAC**
**21**	**RvpMOtag/AEK1**	CTCGAGGGGGGGCCCGGTACCCGGATTTTTATTCAAGTGGTTATC
**22**	**FwAEK1/pMOtag**	GATAACCACTTGAATAAAAATCCGGGTACCGGGCCCCCCCTCGAG
**23**	**FwAEK1_+143**	TGAGAGTCTGGATGTACTTGG
**24**	**RvAEK1_+572**	TCAGCCAAAACACAATGTCC

Bold uppercase: specific protospacer; italic uppercase: restriction site; bold underlined uppercase: gene-specific homologous region in ultramers used for knockout strategies; italic lower case: mismatch nucleotides
